# Burden of tuberculosis among vulnerable populations worldwide: an overview of systematic reviews

**DOI:** 10.1016/S1473-3099(23)00372-9

**Published:** 2023-12

**Authors:** Stefan Litvinjenko, Olivia Magwood, Shishi Wu, Xiaolin Wei

**Affiliations:** a Dalla Lana School of Public Health, University of Toronto, Toronto, ON, Canada; b Bruyère Research Institute, Ottawa, ON, Canada; c Interdisciplinary School of Health Sciences, Faculty of Health Sciences, University of Ottawa, Ottawa, ON, Canada

## Abstract

**Background:**

Tuberculosis is a communicable disease of public health concern that inequitably impacts the most vulnerable populations worldwide. Vulnerable populations are those with a high risk for tuberculosis disease and whose disadvantaged or marginalised socioeconomic position limits their access to the health system. We conducted an overview of reviews that aimed to assess the burden (ie, prevalence and incidence) of tuberculosis disease among 12 vulnerable populations globally.

**Methods:**

We did an overview of reviews using a systematic search in MEDLINE, Embase, and the Cochrane Database for Systematic Reviews for articles published in English, French, and Chinese, from Jan 1, 2010 to March 8, 2023. We did an initial search on Oct 28, 2021, and updated our search on March 8, 2023. We included systematic and scoping reviews reporting on the prevalence or incidence of active tuberculosis among 12 vulnerable populations. Evidence gaps were supplemented with primary or secondary database studies. Study characteristics and outcome data related to tuberculosis burden were tabulated, including prevalence ratios and incidence rate ratios, and evidence was synthesised narratively. This trial is registered with PROSPERO (CRD42022324421).

**Findings:**

We screened 13 169 citations and included 44 publications (23 reviews and 21 primary or database studies) in the final synthesis. The comprehensiveness and methodological quality of the evidence differed across population groups. Prevalence of more than 1000 cases per 100 000 were reported in all vulnerable populations. On the basis of pooled estimates, prevalence ratios were often more than 25 among people experiencing homelessness, incarcerated populations, refugees, asylum seekers, and people living with HIV compared with the general population. Incidence was infrequently reported, with the best-available incidence rate ratios documented for people who were incarcerated. There was scarce evidence specific to miners, nomadic populations, sex workers, men who have sex with men, and transgender individuals.

**Interpretation:**

The burden of tuberculosis is substantially higher among vulnerable populations than general populations, suggesting a need for improved integration of these groups, including dedicated efforts for their identification, targeted screening and prevention measures, as well as treatment support.

**Funding:**

WHO.

## Introduction

Tuberculosis is a communicable disease that is a major cause of morbidity and mortality worldwide. In 2021, an estimated 10·6 million people developed active tuberculosis disease and 1·6 million deaths were caused by tuberculosis.^1^ The COVID-19 pandemic has interrupted the delivery of essential screening and tuberculosis treatment services globally, serving to only further exacerbate underlying inequalities in care. Despite international progress with about a 2% average annual reduction in tuberculosis incidence rates (before 2020) in the general population,^1^ the disease is becoming concentrated among populations with low socioeconomic status, especially vulnerable individuals and those who experience varying forms of social exclusion.^2^ Therefore, tuberculosis is inequitably distributed within and between global regions and is highly influenced by socioeconomic determinants and health-related risk factors,^1^ which can intersect and lead specific population groups to be particularly vulnerable to tuberculosis.

**Figure 1:**
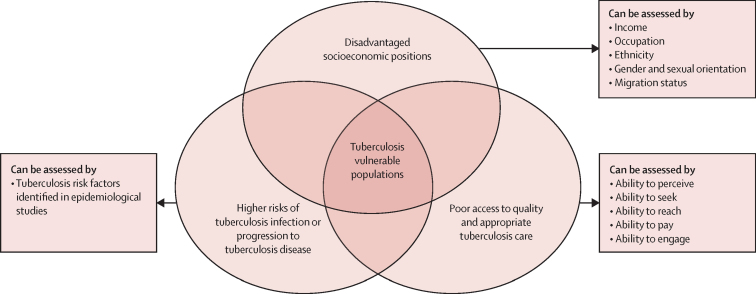
Conceptual model of populations vulnerable to tuberculosis Vulnerability sits at the nexus of socioeconomic positions, access to health systems, and risk of tuberculosis disease, leading to inequitable tuberculosis outcomes. Reproduced by permission from Wu and colleagues.^4^

When referring to people who are at higher risk for infection, disease, or poor outcomes, the term vulnerable is often used interchangeably with other terms including priority, marginalised, disadvantaged, disenfranchised, underprivileged, at-risk, underserved, and hard to reach.^3^ In this review we define vulnerable populations as those who simultaneously hold disadvantaged or marginalised socioeconomic positions or contexts, have higher tuberculosis risks, and have barriers to quality and appropriate tuberculosis care, such as being unaware, unable, or unwilling to seek tuberculosis care and complete treatment.^4^ We propose three dimensions as criteria for identifying vulnerable populations, the socioeconomic positions of individuals, risk of tuberculosis infection and disease, and access to health systems (figure 1).^4^ We differentiate vulnerable populations from at-risk or key populations, which are groups that share a common risk factor or risk exposure or are cohorts of importance on the basis of epidemiological or tuberculosis control considerations, but who might not necessarily lack power or face structural barriers to access health services. For example, health-care workers have an approximately three times greater risk of active tuberculosis than the general population because of their occupational exposure to tuberculosis,^5^ but have fewer delays in tuberculosis diagnosis and treatment and a relatively low percentage of tuberculosis deaths.^6^ Instead, it is the inherent identity, practices, and lived experience of vulnerable populations themselves in the macroenvironment that allow for these groups to have higher risks for tuberculosis, that are in turn associated with later diagnoses, worse treatment adherence, and ultimately poorer health outcomes.^2^

**Research in context**

**Evidence before this study**

We received a list of key documents (policy briefs, guidelines, and a summary of populations included in national tuberculosis strategic plans) from the WHO Global Tuberculosis Programme to inform this review; however scarce data on tuberculosis burden were available for vulnerable populations. Before this study, we did a narrative review to identify and consolidate definitions of vulnerability and establish a list of criteria to identify vulnerable populations to be included. A systematic search in MEDLINE, Embase, and the Cochrane Database for systematic reviews from Jan 1, 2010 to March 8, 2023 using key words related to “tuberculosis” and “vulnerable populations” (eg, “refugees”, “prisoners”, and “homeless”) uncovered studies related to the prevalence and risk of tuberculosis across vulnerable populations. Varying study objectives, methods, and population subgroups were noted, justifying the purpose of a consolidated review. Most studies focused on tuberculosis service delivery and treatment outcomes in vulnerable populations. Current WHO guidance on tuberculosis screening, prevention, and treatment outlines a strong recommendation for screening for tuberculosis in people living with HIV and prison settings (based on very low certainty of evidence). Conditional recommendations pertain to several other vulnerable and marginalised subpopulations because of a scarcity of evidence.

**Added value of this study**

This overview provides a comprehensive global summary of evidence on the burden of tuberculosis in select vulnerable populations. Although previous analyses considered primary studies only, this publication draws comparisons across several pooled estimates (ie, meta-analyses) as a review of reviews. Emphasising robust evidence sources, our work provides insights for where programmatic interventions might have the greatest yield and serves as a framework for countries to define and map their own vulnerable populations to the local context. This review provides data on the burden of tuberculosis in vulnerable populations, rather than broader populations groups such as at-risk people, key populations, and hard-to-reach groups.

**Implications of all the available evidence**

The use and understanding of what constitutes a tuberculosis-vulnerable population is not uniform across tuberculosis programmes, nor among researchers. Vulnerable populations in this overview of reviews experienced universally higher tuberculosis burden in comparison with general populations, although this evidence was distributed unequally among vulnerable groups on a regional and population basis. For well studied groups (eg, incarcerated populations, refugees, and asylum seekers), available estimates highlight areas for priority intervention, and serve as a reference from which tuberculosis programmes can evaluate their own vulnerable groups. Tuberculosis burden remains mostly undefined for several groups from which pooled estimates were not available, revealing opportunities for further research. Surveillance systems require amendments to better capture intersectional vulnerable identities, which have traditionally been challenging to define, find, and capture epidemiological data on. Documenting tuberculosis burden in vulnerable populations and developing tailored programmes to meet their needs is essential to realising their fundamental right to health. Prevention and care models should consider the difficulties faced by vulnerable groups in recognising symptoms, barriers to accessing quality and appropriate tuberculosis care, and treatment-adherence challenges. Research evidence suggests country tuberculosis programmes work with other sectors to develop policies that target the root causes (ie, poverty alleviation), to achieve the Global END Tuberculosis Strategy by 2035.

Current guidance on tuberculosis screening, prevention, and treatment requires revision to better consider the complex and intersecting social identities that contribute to tuberculosis-related vulnerability.^7^ Programme managers and decision makers might benefit from up-to-date evidence on the burden of tuberculosis among vulnerable populations to inform future policy and guidance. This overview of reviews aims to present the breadth of available prevalence and incidence estimates for 12 vulnerable populations and highlight the best available evidence to inform decision making. We address the following research question what are the best available estimates of prevalence and incidence of tuberculosis disease in vulnerable populations worldwide?

## Methods

### Search strategy and selection criteria

We did an overview of reviews according to guidance from Cochrane and reported it according to PRISMA.^8^, ^9^ We searched MEDLINE, Embase, and the Cochrane Database for systematic reviews for articles published in English, French, or Chinese. These languages of publication were selected on the basis of the skills of the review team. We did an initial search on Oct 28, 2021, and updated our search on March 8, 2023. We used a combination of keywords and subject headings to combine concepts of tuberculosis, tuberculosis burden (prevalence and incidence), and vulnerable populations. In addition, grey literature, relevant journals, policy briefs, and technical proceedings (eg, WHO and Stop Tuberculosis Partnership guidelines and documents) were hand searched (appendix pp 2–3).

We included systematic or scoping reviews published between Jan 1, 2010 and March 8, 2023 reporting the prevalence or incidence of tuberculosis disease (ie, active tuberculosis) among any of the vulnerable populations listed (table 1). If none or few eligible reviews were identified for a population, we additionally considered observational (cross-sectional, cohort, and retrospective database) or experimental studies. Detailed inclusion and exclusion criteria are presented in the appendix (p 4).

**Table 1:** Tuberculosis-vulnerable population definitions

	**Definition**
People experiencing homelessness	Adults with no fixed or regular abode (owned or rented) or night-time residence, who rely on temporary accommodation, live in institutions or shelters, the streets, or people who are not living in census houses, but in buildings or in the open on roadsides, pavements, in pipes, under flyovers and staircases, or in the open in places of worship, railway platforms, and other unstable housing situations. Homelessness can be chronic or temporary, voluntary or forced.^10^, ^11^, ^12^, ^13^
Incarcerated populations	Refers to all incarcerated or detained individuals in institutions that hold people who have been sentenced to a period of imprisonment by the courts for offences against the law. This definition includes any form of involuntary detention, including prisons, jails, pretrial centres, immigration detention centres, drug detention or rehabilitation centres, correctional facilities, forced labour camps, and other facilities.^14^, ^15^
Indigenous people	Self-identifying as Indigenous people, with a historical continuity with precolonial or presettler societies, strong links to territories and surrounding natural resources, distinct social, economic, or political systems, distinct language, culture, and beliefs, non-dominant status within society (that might result in disadvantage or discrimination in several areas—eg, success, education, health care, or employment), and the resolve to maintain and reproduce their ancestral environments and systems as distinctive people and communities.^16^, ^17^
People living in slum settings	So-called slum households are those in which inhabitants suffer one or more of the following household deprivations: no or limited access to a potable water source; no access to sanitation facilities; a living area that is not sufficient; no housing durability; and no security of tenure. People living in slums can also be described as those living in informal settlements, which are areas that have been recently urbanised and are overcrowded (plot size 250–800 m^2^) with informal dwellings for a population of low socioeconomic status, or in townships and periurban or resource-limited settings.^18^, ^19^
Refugees, asylum seekers, internally displaced people, and undocumented migrants	A refugee is someone who, owing to well founded fear of being persecuted for reasons of race, religion, nationality, or membership of a particular social group or political opinion, is outside the country of his or her nationality and is unable or, owing to such fear, is unwilling to avail him or herself of the protection of that country. Asylum seekers are people who claim to be admitted to a country as refugees and are awaiting the decision of the authorities. Internally displaced people are individuals who have been forced or obliged to flee or to leave their homes or places of habitual residence to avoid the effects of armed conflict, violence, violations of human rights, or natural or human-made disasters, and who have not crossed an internationally recognised border.^20^, ^21^
Miners	Includes active miners working in mining sites in the formal (regulated) sector, and informal (artisanal) mining settings that might entail the services or support provided by non-officially employed or registered people in mining communities at large. Activities vary on the basis of the commodity collected (eg, gold, copper, or coal).^19^, ^22^, ^23^
Nomadic populations	Mobile, or non-settled communities of people who constantly migrate in search of pasture for their livestock, subsisting on hunting and gathering or often driven by climatic conditions. Such communities usually roam over hundreds of kilometres, and often pass through resting points where temporary tents are erected for the purpose of resting the herds and seeking medical attention for both people and animals.^24^
Sex workers	Individuals who receive money, goods, or services for providing sex services (on a regular or irregular basis). These people are typically considered people born female, but sex workers are not limited to a single gender. The individuals are sometimes referred to as commercial sex workers.^13^, ^25^
Men who have sex with men	Defined as born male, men who practice sexual contacts (oral or anal) with another person born male.^13^, ^25^
Transgender individuals	A person whose gender identity, expression, or behaviour does not correspond with their assigned sex at birth. Inclusive of transgender women and transgender men.
People who use drugs	A person who consumes large and regular doses of (possibly different) drugs, resulting in addiction (ie, the loss of capability to refrain from using the drugs, progressively affecting social life, material status, and existence in general), or attendance at medication-assisted treatment clinics. People who inject drugs are individuals who regularly inject narcotic drugs with non-therapeutic purposes.^2^, ^13^
People living with HIV	Individuals living with HIV, irrespective to whether patient status is known or not and an official diagnosis has been registered. Most commonly confirmed through a seropositive test result within 3–12 weeks of contraction of the virus.

Vulnerable populations as identified by Wu and colleagues.^4^

We used a pragmatic selection method adapted from guideline development methods, selecting the most comprehensive and recent evidence sources,^26^ often termed the best-and-brightest approach.^27^ Following a piloting exercise, four independent reviewers (SL, Weilin Zhang, Krittika Bali, and Betty Li) screened the titles and abstracts of all identified citations in duplicate against our eligibility criteria using Covidence software.^28^ In our initial search, two reviewers (SL and OM) selected a sample of eligible studies at full text and achieved good agreement, with the remainder selected by one reviewer (SL). In our search update, all studies were assessed for eligibility by two reviewers (SL and OM), independently and in duplicate. The two reviewers agreed on the final set of publications eligible for inclusion, and disagreements were resolved through discussion with a third review author (XW). We prioritised recent and comprehensive systematic and scoping reviews which offered global or regional estimates of tuberculosis disease.^27^

### Data analysis

Data were extracted by one review author (SL) and verified, independently, by a second review author (OM). For included reviews, we extracted the population definition, number and date range of included studies, population sample size, and countries and regions from which data were reported. Overlapping studies within vulnerable groups were assessed, and study characteristics (eg, sample sizes) were tabulated and assigned to the most comprehensive report only, including effect estimates for non-pooled analyses, to prevent double counting. For included primary studies, we extracted the study design and length of follow-up, setting and country, population eligibility criteria and sample characteristics, data sources, and tuberculosis diagnostic approaches. For outcome data, we extracted measures of prevalence and incidence including any subgroup analyses. We prioritised pooled estimates over single-study or single-country estimates. When available, we extracted comparisons with general populations as prevalence ratios or incidence rate ratios. We assessed the methodological quality of included reviews using A Measurement Tool to Assess Systematic Reviews, version 2 (AMSTAR 2).^29^ For included observational studies, we assessed quality using a modified version of the Joanna Briggs Institute (JBI) checklist for prevalence studies.^27^ All assessments were completed by one reviewer and verified by a second reviewer. Disagreements were resolved through discussion.

We synthesised outcome data narratively.^30^ We organised the presentation of relevant study characteristics and evidence by vulnerable population groups. We tabulated measures of prevalence and incidence as primary outcome data and recorded the presence of subgroup data or analyses (and their contribution to tuberculosis burden). Cases per 100 000 population was used as a standard metric, and we transformed source data to this format when required. We included existing meta-analyses with pooled effects where appropriate, and report estimates of effect and CIs as reported in the source review. We use the comparisons as reported in the source review. To visually present the data, ad-hoc analyses were done using Microsoft Excel software (version 16.45), including mapping evidence availability by country, and displaying effect estimates on a forest plot (without pooling). This review was registered in PROSPERO (CRD42022324421).

### Role of the funding source

The funder of the study had no role in study design, data collection, data analysis, data interpretation, or writing of the report.

## Results

**Figure 2:**
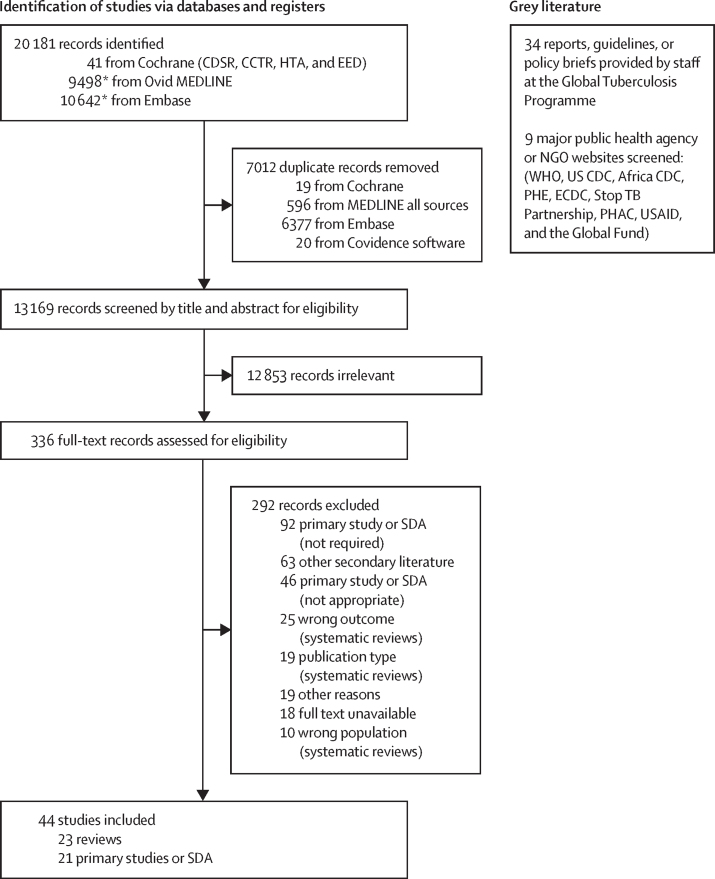
PRISMA flow diagram PRISMA 2020 flow diagram for new systematic reviews, which included searches of databases, registers, and other sources. Definitions for exclusion reasons are shown in the appendix p 5. CCTR=Cochrane Central Register for Controlled Trials. CDSR=Cochrane Database for Systematic Reviews. CDC=Centers for Disease Control and Prevention. ECDC=European Centers for Disease and Control. EED=National Health Service Economic Evaluation Database. HTA=Health Technology Assessment Database. NGOs=non-governmental organisations. PHAC=Public Health Agency of Canada. PHE=Public Health England. SDA=secondary analysis (database) studies. USAID=US Agency for International Development. *106 qualifying records from Embase and 102 records from MEDLINE were identified in the French or Chinese language. Among these records were 48 duplicates (all from Embase). None of these citations advanced past the title or abstract screening stage.

Our systematic search identified 20 181 citations. After removal of duplicates, we screened 13 169 citations by title and abstract against a-priori inclusion criteria. Of these, 336 were retained for full-text assessment, and 292 were excluded (appendix p 5). In total, we included 44 publications (figure 2). No additional publications were included from the grey literature.

Among the 44 included publications, we included 23 reviews and an additional 21 studies not captured within existing review evidence. These publications reported on people experiencing homelessness (n=4), incarcerated populations (n=10), Indigenous people (n=5), populations living in slum settings (n=1), refugees, asylum seekers, and internally displaced people (n=5), miners (n=4), nomadic populations (n=3), men who have sex with men, transgender women, and female sex workers (n=1), people who use drugs (n=3), people living with HIV (n=1), and mixed vulnerable populations (n=7). There was no systematic review evidence available for nomadic populations, men who have sex with men, and sex-worker populations, and evidence for several groups (eg, miners and people who use drugs) came from a scarce number of studies or countries. We visually presented the geographical availability of evidence in a series of maps (appendix pp 24–28). A full account of the characteristics of all included studies and outcome data are listed in the appendix (pp 6–19). The heterogeneity of prevalence and incidence estimates are displayed in a series of non-pooled forest plots, which were established as the data permitted (appendix pp 20–23). A summary of the best available estimates organised by population group is presented in table 2.

**Table 2:** Summary of characteristics of included studies and best-available estimates on tuberculosis burden in vulnerable populations

	**Included studies**	**Studied population: sample size, number of studies, and countries**	**Date range of evidence**	**Pooled prevalence per 100 000 (95% CI)**	**Range of prevalence per 100 000**	Prevalence ratio*	**Incidence per 100 000 person-years**	Incidence rate ratio or alternative measure of association*
People experiencing homelessness	Three systematic reviews, one database study, two primary studies and one database study on mixed groups†	Population of at least 142 000; US registry data; 33 studies in 16 countries	1980–2020	1100 (800–1500) from 17 studies^10^	200–7700 from 17 studies^10^	Range of 22·4 (Sweden) to 461·2 (USA) from 17 studies^10^	Range 31 (USA)^12^ to 900 (Romania)^2^, ^31^	10·7 (USA)^12^ and 12·8 (Romania)^2^
Incarcerated populations	Nine systematic reviews, one database study, two systematic reviews on mixed groups,† one scoping review on mixed groups,† two primary studies, and one database study on mixed groups†	Unspecified total population; 53 countries represented; European registry data; and based on the largest review: more than 6 700 000 people (prevalence) from at least 106 studies;^14^ more than 1 850 000 people (incidence) from at least 51 studies^14^	1980–2021	Overall: 2371 (1983–2759) from 22 studies^32^ to 2790 (2050–3650)^15^, ^33^ with study number not reported; by region:^14^ North America 320 (130–650) from 12 studies, South America 1680 (830–2970) from 16 studies, Europe 1000 (510–1770) from 18 studies, Africa 1610 (980–2500) from 30 studies, southeast Asia 1810 (670–4000) from eight studies, western Pacific 720 (270–1600) from ten studies, and eastern Mediterranean 1160 (480–2370) from 11 studies	Unspecified range	Overall:^32^ 25·2 (95% CI 11·6–38·8); by region:^32^ Middle East 3·4 (2·4–4·4); Asia 4·3 (2·0–6·7); South America and central America 49·9 (32·1–67·7); Africa 1·9 (0·5–3·2)	Estimates are pooled estimates;^14^ Overall: not reported; by region: North America 30 (95% CI 20–50) from 12 studies; South America 970 (460–1860) from 12 studies; Europe 610 (310–1100) from 11 studies; Africa 2190 (810–4840) from seven studies; southeast Asia 1550 (240–5300) from three studies; western Pacific 390 (80–1130) from three studies; eastern Mediterranean 270 (50–880) from three studies	Estimates are pooled estimates;^14^ Overall: 10·1 (95% CI 7·6–13·0) from 47 studies; by region: North America 4·1 (2·8–6·2) from seven studies; South America 26·9 (17·1–40·1) from 12 studies; Europe 8·7 (3·7–16·8) from six studies; Africa 12·6 (6·2–22·3) from six studies; southeast Asia 11·7 (4·1–27·1) from three studies; western Pacific 6·8 (2·9–13·2) from three studies; eastern Mediterranean 15·6 (6·5–32·5) from three studies
Indigenous people	Two systematic reviews, two database studies, and one primary study	Unspecified total (at least 412 000); registry data in the USA and Brazil; 113 studies in 22 countries	1990–2020	By region:^17^ southeast Asia 2200 (1600–3000) from 18 studies and western Pacific 2300 (700–4900) from six studies	By region:^16^ Africa 1800 (for the Peul and Dogon people in Mali) and 4600 (for the nomadic Fulani in Chad) from two studies; Latin America and Caribbean 211 (the Xavante in Brazil) to 6700 (Indigenous people in Ecuador) from eight studies; Europe 85 (Inuit people in Greenland) to 1160 (Indigenous people of Chukotka Autonomous District, Russia) from four studies; southeast Asia 100 (male Gond Gawari people in India) to 46 197 (people of the Saharia tribal groups in India) from 17 studies; western Pacific 47 (Bai people in China) and 395 (ethnic minorities that were not specified in China) from two studies	Comparator population;‡^16^ range of 5·5 (Peul and Dogon people in Mali) to 10·7 (the nomadic Fulani in Chad) from two studies; Latin America and Caribbean, range of 3·8 (the Xavante in Brazil) to 94 (Yanomami people in Brazil Amazonas) from eight studies; Europe, range of 0·9 (Indigenous people in Russia) to 15·3 (Inuit people in Greenland) from four studies; southeast Asia, range of 0·5 (Baiga and Jawadhu–Hills tribes in India) to 138 (the Saharia tribal groups in India) from 17 studies; western Pacific 0·4 (Bai people, China) to 3·1 (ethnic minorities that were not specified, China) from two studies	Regional:^16^ North America, range 4·3 (American Indian and Alaska Native, USA) to 431 (Indigenous, Canada) from 23 studies; Latin America and Caribbean, range 42·3 (Aguaruna, Peru) to 3700 (Ache Natives, Paraguay) from 11 studies; eastern Mediterranean: 18·1 (Bedouins, Israel) from one study; Europe, range 85 to 304 (Chukotka Autonomous Indigenous, Russia) from four studies; southeast Asia, range 770 (Tibetan refugees, India) to 8377 (Tibetan refugees, USA) from four studies; western Pacific, range 0·8 (Aborigines and Torres Strait Islander descendants, Australia) to 6500 (E–Lun Chun, Ortochen, China) from 16 studies	Comparator population;‡^16^ North America, range 0·3 (Yukon, Canada) to 331 (Saskatchewan, Canada) from 23 studies; Latin America and Caribbean, range 0·4 (Shapra, Peru) to more than 1000 (Indigenous, Brazil) from 11 studies; eastern Mediterranean 2·1 (Bedouins, Israel) from one study; Europe, range 2·3 (Chukotka Autonomous Indigenous, Russia) to 10 (Indigenous, Russia) from four studies; southeast Asia, range 3·6 (Tibetan refugees, India) to 762 (Tibetan refugees, USA) from four studies; western Pacific, range 0·6 (Aborigines and Torres Strait Islander descendants, Australia) to 42·5 (E–Lun Chun, Ortochen, China) from 16 studies
People living in slum settings	One systematic review, one systematic review on mixed groups,† and one scoping review on mixed groups†	Unspecified total; more than 2 420 000 (incidence) from at least 20 studies; unspecified total; more than 840 000 (prevalence), from at least eight studies; 31 studies in 13 countries	1966–2018	Not reported	45·8 (India) to 3548 (Uganda) from eight studies^18^, ^19^, ^32^	Range 1·6 (Philippines) to 5·5 (Uganda) from three studies^18^	Range 5·1 (India) to 8824 (Peru) from 20 studies^18^, ^19^	Pooled odds ratio compared with national rates 3·0 (95% CI 2·8–3·1) from 22 studies^18^
Refugees, asylum seekers, and internally displaced people	Four systematic reviews on refugees or asylum seekers, one systematic review and one scoping review on mixed groups† with refugee data, and two primary studies on internally displaced people (one study from mixed groups)†	At least 470 000 refugees and 340 000 asylum seekers, and more than 200 000 internally displaced people; 78 studies from 23 host countries or countries of study	1980–2021	Refugee and asylum data combined; by country of origin:^20^ 1331 (542–2384) from 16 studies; by host continent:^20^ Americas 1080 (405–2035) from three studies, Asia 860 (0–3588) from three studies, Europe 1458 (570–2648) from 12 studies; refugees:^34^ 1192 (678–1717) from seven studies; asylum seekers^34^, ^35^ 304 (224–367) from 13 studies	Refugees (by country of origin): 0 (Bhutan, Burma) to 11 364 (Syria) from 28 studies;^20^ asylum seekers (by country of origin): 69 (not reported) to 6250 (Afghanistan, Armenia, Eritrea, Nigeria, and Syria) from 13 studies;^20^ internally displaced people 317 (95% CI 80–1756; Ethiopia)^36^ to 598 (camp settings in Nigeria)^37^	Compared to autochthonous population:^34^ refugees 130·6 (95% CI 58·8–290·2) from seven studies; asylum seekers 30·1 (19·3–47·1) from seven studies; internally displaced people in Nigeria, 2·3^37^; internally displaced people in Ethiopia, 1·9^36^	Not reported	Not reported
Miners	One systematic review on mixed groups,† one scoping review on mixed groups,† four primary studies, and two primary studies on mixed groups†	At least 125 000; 17 studies in seven countries (mostly South Africa)	1966–2022	Not reported	1300 (South Africa) to 13 512 (Malawi) from 14 studies^19^, ^22^, ^23^, ^36^, ^38^, ^39^, ^40^	Range 7·7 (Ghana) to 40 (Malawi) from five studies^22^, ^23^, ^36^, ^39^, ^40^	3000 (South Africa)^19^	Relative risk 3·8 (South Africa)^19^
Nomadic populations	Three primary studies and one primary study on Roma people among mixed groups†	At least 102 000 (excluding Roma people, because the total could not be reliably tallied); data limited to four single-country primary studies	2012–18	Not reported	73 (Iran) to 2000 (Mauritania) from four studies^13^, ^24^, ^41^, ^42^	4·2 (Nigeria) to 9·9 (Mauritania) from three studies^24^, ^41^, ^42^	Not reported	Not reported
Sex workers, men who have sex with men, and transgender individuals	One primary study, three primary studies on mixed groups,† and one systematic review on incarcerated transgender individuals	9759 female sex workers, 863 men who have sex with men and transgender women, 199 incarcerated transgender women; data limited to six single-country primary studies	1996–2020	Not reported	Female sex workers: 200 (95% CI 0–500; Papua New Guinea) to 2817 (1678–4415; Ethiopia) from three studies;^25^, ^36^, ^40^ men who have sex with men and transgender women: 1000 (0–2200) to 1200 (0–2400; Papua New Guinea) from one study^25^	Female sex workers: 0·6 (Papua New Guinea) to 17·2 (Ethiopia) from three studies;^25^, ^36^, ^40^ men who have sex with men and transgender women: 3–3·6 (Papua New Guinea) from one study^25^	Not reported	Not reported
People who use drugs	Three primary studies, one systematic review on mixed groups,† one primary and database study each on mixed groups,† and two systematic reviews with subpopulation data on people who inject drugs (incarcerated)	Unspecified total; at least 3839 individuals across 14 studies in at least eight countries	2001–19	Not reported	2100 (95% CI 800–4200; Viet Nam) to 9793 (Ivory Coast) from three studies;^43^, ^44^, ^45^ and in incarcerated people who inject drugs 0–24 500 from five studies^14^, ^15^	48·9 (Ivory Coast)^44^	630 (Romania)^2^ and 2925 (95% CI 2195–3958; Tanzania);^45^ incarcerated people who inject drugs: 1240–7975 from two studies^14^	8·9 (Romania)^2^ and 12·3 (Tanzania)^45^
People living with HIV	One meta-review encompassing five systematic reviews, two systematic reviews and one scoping review on mixed groups,† two systematic reviews on incarcerated people living with HIV, and subgroup co-infection data across numerous vulnerable population groups	Unspecified total; at least 388 000; unspecified number of studies in 35 countries	1985–2019	Overall: ^46^ 23 500 (20 900–26 100) from 47 studies; HIV among people with tuberculosis: 25 200 (18 600–31 900) from 29 studies; tuberculosis among people with HIV: 23 300 (16 700–29 900) from 11 studies; by region: Africa 31 300 (19 300–43 200) from 17 studies, Asia 17 200 (10 000–24 500) from nine studies, Latin America 25 100 (19 300–30 800) from seven studies, Europe 20 100 (13 800–26 400) from eight studies, USA 14 800 (10 400–19 200) from six studies	Based on the largest review: 2900 (Iran) to 72 000 (Zambia) from 47 studies^46^	Overall:^32^ 19·7 (95% CI 13·7–25·7); by region:^32^ North America and Europe: 17·4 (9·7–25·2); Asia 25·6 (19·3–31·9)	Not reported	Not reported

Best-available summary statistics were selected on a pragmatic basis for display purposes only. Precedence was given to systematic reviews or meta-analyses that considered the largest study sample size, had the widest geographical reach, and were most recent. A full list of results is presented in the appendix (pp 6–9).

* Unless otherwise stated, comparator values refer to corresponding general population prevalence or incidence rates (typically at the national level, per 100 000 people). This information was not always specified in source material. Reference values did not always correspond to the year in which studies were done. Comparators were used as presented by source authors, or where absent were calculated manually as permitted.

† Several reviews or studies screened mixed populations within a single publication. This commonly refers to the study or reporting of several (separate) groups within a single review, or related, but distinct populations grouped under one heading (eg, refugees and immigrants or poor and marginalised). Where possible, we extracted data into individual vulnerable groups and marked these studies accordingly to prevent their repeated tally as unique records.

‡ Tollefson and colleagues (2013):^16^ the comparison groups used in studies differed widely between studies. Numbers provided by authors for regional estimates were sometimes regional averages, whereas other times they were non-indigenous tuberculosis rates within those regions.

After critical appraisal using AMSTAR-2, ten of 23 reviews scored critically low, ten of 23 scored low, one of 23 scored moderate, and two of 23 scored high (appendix pp 29–30). Although observational studies inherently have some risk of bias, the 21 primary-database studies included in our review scored well using the JBI checklist for prevalence studies (appendix pp 31–32). In people experiencing homelessness, three systematic reviews provided global data but the majority of studies came from high-income countries, particularly from Europe and the USA.^10^, ^47^ The pooled prevalence of tuberculosis was 1100 cases (95% CI 800–1500) per 100 000.^10^ Additional reviews provided prevalence data (per 100 000) from Japan (n=1400), Brazil (n=2800), Ethiopia (n=2600), and the USA (n=6000).^31^, ^47^ Incidence rates were obtained from primary or database studies, ranging from 31 cases to 900 cases per 100 000, and were scarce outside of the USA.^2^, ^12^, ^31^, ^47^ Prevalence ratios ranged from 22·4 (Sweden) to 461·2 (USA) on the basis of a single review.^10^

Global tuberculosis prevalence in incarcerated populations was estimated to range between 2371 cases (95% CI 1983–2759)^32^ to 2790 (2050–3650) per 100 000.^15^, ^33^ Several meta-analyses provided estimates by global region,^14^, ^15^, ^32^ with one review reporting a prevalence ratio of 49·9 (95% CI 32·1–67·7) in South and central America, several times higher than other regions.^32^ On the basis of a review of nearly 7 million people, pooled tuberculosis prevalence was greater than 1000 cases per 100 000 incarcerated people in all regions except North America and the western Pacific.^14^ Pooled tuberculosis incidence (cases per 100 000 person-years) varied regionally, ranging from 30 cases (95% CI 20–50) in North America, to 2190 cases (810–4840) in Africa.^14^ Globally, the incidence rate ratio was 10·1 (95% CI 7·6–13·0) and was highest in South America 26·9 (17·1–40·1).^14^ An earlier review reported a median incidence rate ratio of 23·0 (IQR 11·7–36·1).^48^ Adolescents and young adults younger than 25 years who are incarcerated were not at a statistically significant higher risk for tuberculosis than older incarcerated persons.^49^ The pooled prevalence of tuberculosis and HIV co-infection in incarcerated people was 32·6% (95% CI 27·5–38·2).^50^

The prevalence and incidence of tuberculosis among Indigenous people worldwide varied by region and group. Evidence came primarily from small studies included in a review that reported data regionally by tribal group without pooled estimates^16^ (appendix p 10). The prevalence of tuberculosis in Indigenous people ranged from 85 cases to 6700 cases per 100 000, with the exception of the Forest people in India (n=18 000), and the Saharia (n=46 197), who also remained the most disadvantaged when compared to the general population (prevalence ratio 138, 95% CI not available).^16^ A single pooled prevalence estimate came from a review in the southeast Asia and the western Pacific region (2300 per 100 000, 95% CI 1700–2900).^17^ Incidence rates among Indigenous people in high-income countries were lower than in other Indigenous groups worldwide, however these groups had high incidence rate ratios (eg, 331, 95% CI not available, in Saskatchewan, Canada,^16^ and 20·1, 95% CI 13·9–28·9 among Native Hawaiians or other Pacific Islanders in the USA).^51^ Data from the African and eastern Mediterranean regions were scarce across all metrics.

A single review on people living in slum settings provided incidence data for more than 2 million individuals.^18^ Incidence per 100 000 ranged from five cases to 8825 cases, with incidence rate ratios of up to 58 reported.^18^ Limited prevalence estimates were noted from single countries.^18^ The best-available estimates from a review in South Africa reported a prevalence of 3150 and incidence of 4500 per 100 000 people living in informal settlements.^19^ A few unique estimates were also reported in a review of populations that lived in poverty and were marginalised.^32^

Among refugees and asylum seekers, the pooled tuberculosis prevalence was 1331 cases (95% CI 542–2384) per 100 000.^20^ By host continent, this was 1458 (95% CI 570–2648) in Europe, 1080 (405–2035) in the Americas, and 860 (0–3588) in Asia.^20^ The greatest number of studies were on refugees and asylum seekers arriving to Europe, allowing for the assessment of heterogeneity by country of origin.^34^, ^35^ Assessing populations individually, refugees had a nearly four-times greater screening yield upon entry (1192 per 100 000, 95% CI 678–1717)^34^ than asylum seekers (304 per 100 000, 224–367).^35^ Compared with the general populations, refugees had a prevalence ratio of 131 (95% CI 59–290) and asylum seekers of 30·1 (19·3–47·1).^34^ Scarce data on internally displaced people came from primary studies in Ethiopia (317 per 100 000, 95% CI 80–1756)^36^ and Nigeria (502 per 100 000, 95% CI not available).^37^ No evidence was identified for undocumented migrants.

No systematic reviews were identified specific to miners. Limited mixed-group reviews provided data from South Africa^19^, ^38^ and other primary studies covered heterogeneous study populations and different forms of mining spanning six African countries. The prevalence of tuberculosis ranged from 1300 cases to 13 512 cases per 100 000, with prevalence ratios of 7·7–40 reported.^19^, ^22^, ^23^, ^39^

Similarly, reviews were not identified for nomadic populations. Available primary studies were based in the African^24^, ^42^ or Middle Eastern^41^ context, and evidence from Ukraine suggests increasing incidence of tuberculosis among Roma populations.^13^ The prevalence of tuberculosis in nomads varied from 73 cases to 2800 cases per 100 000 (95% CI 1500–4700). Prevalence ratios were between 4·2 and 14.^24^, ^41^, ^42^

Data on sex workers, men who have sex with men, and transgender individuals was limited to six primary studies.^13^, ^25^, ^36^, ^40^, ^52^ The majority of sampled people were female sex workers, with tuberculosis prevalence ranging from 200^25^ cases per 100  000 (0–500) to 2817 cases per 100 000 (1678–4415),^36^ with prevalence ratios of 0·6–17·2.^25^, ^36^, ^40^ A single estimate among men who have sex with men and transgender women reported a prevalence of 1000–1200 (prevalence ratio 3·3–3·6),^25^ in addition to 0–34 cases per 100 000 in men who have sex with men in Ukraine.^13^ Beyond inconclusive questionnaire data,^52^ no subgroup review data was found for incarcerated female sex workers, men who have sex with men, and transgender people.^15^

A few primary studies provided estimates on tuberculosis prevalence in people who use drugs, including a pooled value of 1157 cases per 100 000 (95% CI 809–1505) based on two studies (prevalence ratio 11·9, 95% CI 11·1–12·7).^32^ Higher rates were noted in Tanzania and the Ivory Coast (2553 cases per 100 000 in Tanzania and 9793 cases per 100 000 in the Ivory Coast, prevalence ratio 48·9, 95% CI not available).^44^, ^45^ For people who inject drugs, data were available from Viet Nam and Tanzania, with a reported prevalence of 2100 cases per 100 000 (800–4200) for Viet Nam and 4000 cases per 100 000 (prevalence ratio 23) for Tanzania, in addition to variable prevalence rates (200–1400) among a large sample in Ukraine.^13^, ^32^, ^43^ Burden in incarcerated people who inject drugs could not be pooled because of heterogeneity (prevalence 0–24 500 per 100 000, n=5 studies;^14^, ^15^ incidence 1240–7975 per 100 000 person-years; n=2 studies).^14^

One meta-review cited several systematic reviews reporting on the prevalence of tuberculosis and HIV co-infection.^46^ On the basis of data from 21 countries, the estimated prevalence was 23 510 cases per 100 000 (95% CI 20 910–26 110) and ranged from 2900 cases to 72 000 cases per 100 000.^53^ Regional pooled prevalence per 100 000 was highest in Africa (31 250, 95% CI 19 300–43 170) and ranged from 15 000 cases to 25 000 cases per 100 000 in other regions.^53^ In sub-Saharan Africa the pooled prevalence rate was 31 810 (27 830–36 070) and was specifically highest in southern and central sub-Saharan Africa.^54^ Lower estimates among people living with HIV were reported in a separate review, corresponding to the only available prevalence ratios of 17·4 (95% CI 9·7–25·2) in North America and Europe, and 25·6 (19·3–31·9) in Asia.^32^

## Discussion

Tuberculosis continues to affect vulnerable groups who have high risks of exposure to the disease and who have social barriers that limit their access to timely high-quality tuberculosis care. As such, the WHO END Tuberculosis Strategy calls for the strengthening and expansion of core functions of tuberculosis programmes, including outreach to vulnerable populations.^55^ This strategy requires not only the systematic definition and identification of vulnerable groups in the national context, but also an estimation of their burden of tuberculosis disease. Our overview of reviews reaffirms a high prevalence of tuberculosis among vulnerable populations such as those experiencing homelessness, incarcerated populations, Indigenous people, and refugees and asylum seekers, often more than 25 times higher than the general population.^10^, ^14^, ^16^, ^34^

Of note, this review specifically addresses populations considered vulnerable to tuberculosis from a social and equity perspective,^4^ as opposed to a wider set of at-risk or key populations, which are often included in the tuberculosis literature on the basis of biomedical, clinical, or epidemiological risks.

We observed notable differences in the quality of evidence documented between and within tuberculosis-vulnerable populations. Pooled estimates were available for less than half of all groups studied, and were comprehensively reported for incarcerated individuals, people living with HIV, refugees, and asylum seekers. Aside from people living with HIV, who have substantial tuberculosis co-infection rates, the pooled prevalence of tuberculosis was highest among incarcerated populations at roughly 2500 cases per 100 000, and similar estimates were reported among Indigenous populations in southeast Asia and western Pacific regions. We have documented absolute prevalence rates greater than this value; however, these estimates are often based on crude values that do not have CIs or were presented as a range in the absence of reliable methods for meta-analysis. To this regard, a small number of primary studies formed the basis of review evidence for miners and people who use drugs, whereas no reviews were identified for nomadic populations, men who have sex with men, and sex workers. Accordingly, there is limited certainty as to which inferences can be drawn from these groups.

Less comprehensive review evidence covered populations such as people experiencing homelessness and Indigenous people, which we supplemented with national database studies. In these groups, evidence predominantly originated from high-income countries, which are typically countries with low tuberculosis burden.^10^, ^16^ This finding suggests that other factors such as within-country social inequality or health-related behaviours might influence the burden of tuberculosis among vulnerable groups more than tuberculosis endemicity. Indeed, the prevalence and incidence of tuberculosis will vary substantially depending on context, including geographical, cultural, sociopolitical, and biomedical factors. For example, Cormier and colleagues^56^ reported that illicit drug use, food insecurity, and tobacco consumption were the most prevalent determinants associated with tuberculosis in Indigenous populations globally, alongside diabetes and alcohol use in high-income countries.

We also found that refugees had a four-times greater increase in tuberculosis prevalence than asylum seekers, and prevalence ratio of 130 compared with autochthonous populations, and that the prevalence of tuberculosis among asylum seekers was similar to that of general immigrants (eg, international students and economic migrants).^34^ Similarly, high prevalence ratios (49·9) and incidence rate ratios (26·9) documented among incarcerated populations in South America provide insights as to where tuberculosis control initiatives might have the most impact.^32^ Comparable estimates in Africa or the eastern Mediterranean should not be discounted, given that countries with poor surveillance capacities might underreport the true burden.^14^, ^15^, ^57^

Incidence data were universally limited across vulnerable populations, even in historically well studied groups. Congregate settings such as prisons, homeless shelters, and slum dwellings represent opportunities for active case finding when compared with vulnerable populations in the wider community who might be difficult to define, identify, and successfully engage. Nevertheless, even in congregate settings, estimating incidence was reportedly challenging because of the dynamic, transient nature of these environments, the calculation of person-time at risk, and the exclusion of prevalent tuberculosis cases that are not always documented at entry.^58^

Vulnerable groups had to be a systematically defined population to facilitate their study, which meant that we excluded ecological studies (eg, the study by de Paiva and colleagues)^59^ that described general correlations of tuberculosis incidence with social vulnerability index measures such as human capital, urban infrastructure, income, and Gini coefficients. Such findings were beyond the scope of this review but are valuable in considering the connection between social determinants of health and tuberculosis-related vulnerability. Subgroup analyses of included studies have occasionally highlighted overlapping risk profiles (or identities; appendix pp 6–19), denoting that membership between vulnerable populations is not mutually exclusive.^2^ Nevertheless, the comprehensive collection of vulnerable population characteristics within national tuberculosis surveillance systems is not currently routine, standardised, or widespread.

As an overview of reviews, to our knowledge this is the most comprehensive assessment of the burden of tuberculosis in several vulnerable populations. Systematic reviews and meta-analyses were used as a base unit of measure, ensuring the highest quality of evidence available, and using the year 2010 as a publication cutoff meant only the latest evidence was considered. The quality of included reviews scored low to critically low in all but three instances, which reflects the rigidity of appraisal tools rather than the inherent quality of some publications. Additionally, where evidence gaps were noted, or robust primary studies or secondary data analyses were available, we supplemented our results with more recent literature.

Several limitations were noted in this review. Studies incorporated into reviews were done for different reasons using various study designs, methods, and case definitions. For example, some studies reported active tuberculosis as a main unit of measurement (presumably inclusive of both pulmonary and extrapulmonary tuberculosis cases), whereas others explicitly monitored pulmonary tuberculosis. This subtle difference can have a substantial effect on estimates considering that 15–25% of all tuberculosis cases are extrapulmonary.^1^ Logistical differences in tuberculosis screening algorithms,^32^ using definitive (microbiologically confirmed) or presumptive (clinical or imaging) diagnostic methods,^48^ the local context, or population characteristics preclude reliable comparisons in the data. This limitation was particularly true for miners, with a heterogeneity of activities (eg, gold, copper, or coal mining) and settings (formal or informal mining) reported, each of which entail varying tuberculosis risks and occupational health supports (or absence thereof).

Furthermore, we excluded single-site studies and studies not published in English, French, or Chinese, potentially biasing against the inclusion of evidence from less studied populations and areas representative of countries with high tuberculosis burden. The decision to exclude tuberculosis infection is likely to have contributed to these findings and might serve as an entry point for future studies on tuberculosis-related vulnerability. In addition, our evidence is representative of the published, peer-reviewed literature, which offers incomplete representation of programmatic efforts to target tuberculosis in vulnerable populations. Linkages between tuberculosis-programme registries and third-party databases have shown utility in being able to document tuberculosis burden in vulnerable populations and these potentials should be further developed.^12^, ^60^

Notably, our review did not involve any novel statistical analyses but rather consolidates and reports on a wide range of available estimates for vulnerable populations to inform decision making. In our interpretations of the evidence, we emphasised pooled estimates over single-study reports of prevalence and incidence. However, we did not formally adjust for population weights or other characteristics of studies in the meta-analyses of burden in the included systematic reviews. Therefore, the estimates we presented should be interpreted with reservations. Programme managers should aim to collect or consult local data for vulnerable groups relevant to their context to inform programmatic changes.

Lastly, our review was not optimised to account for age in selecting vulnerable populations. We acknowledge that certain demographic subgroups such as children or older people might qualify as a vulnerable population under country conditions. Children might have limited autonomy in managing their own health, but a child born in a high-income country with low tuberculosis incidence and universal health-care access would not be considered vulnerable on the basis of our definition. In these circumstances, it is often geographical context or other social determinants (eg, Indigeneity or comorbidities) that dictates tuberculosis vulnerability.

We suggest that tuberculosis health-system strategies might work with other sectors to include links with poverty-alleviation strategies and other social protection interventions with the aim to address the underlying social determinants of health that contribute to tuberculosis vulnerability.^61^, ^62^ An initial step is systematically defining vulnerable groups and measuring their tuberculosis burden or risk to inform targeted and sensible interventions. These efforts can be seen by a growing list of national insights on the effectiveness of active case finding across an array of vulnerable populations,^2^, ^13^, ^19^, ^36^ serving as a model for other countries to adopt similar and further strategies. Civil society groups and informed community members are complementary to tuberculosis programmes regarding direct contact and mapping of vulnerable groups, the mobilisation of people or information, creating a demand for care, and framing effective delivery models.^55^ Multisectoral coordination between national governments, NGOs, and industry are essential to developing mechanisms that safeguard economic, legal, and human rights, and to reduce underlying vulnerabilities.^63^, ^64^, ^65^ Our review promotes visibility to the important inequities faced by tuberculosis-vulnerable populations, highlighting that the documentation of tuberculosis burden is not equivalently saturated among vulnerable groups, and both population and regional differences exist. We call for national tuberculosis programmes to define their own vulnerable groups indicated by our study and implement strategies for the explicit integration of vulnerable groups into national surveillance systems, tuberculosis prevention programmes, and care policies.

## Data sharing

All data collected for this study are available in the previously published materials referenced in this manuscript. The study protocol is publicly available on PROSPERO (CRD42022324421). No other material is available from the authors.

## Declaration of interests

We declare no competing interests.
