# Correction to *Lancet Infect Dis* 2023; published online Sept 8. https://doi.org/10.1016/S1473-3099(23)00372-9

**DOI:** 10.1016/S1473-3099(23)00625-4

**Published:** 2023-11

*Litvinjenko S, Magwood O, Wu S, Wei X. Burden of tuberculosis among vulnerable populations worldwide: an overview of systematic reviews.* Lancet Infect Dis *2023; published online Sept 8. https://doi.org/10.1016/S1473-3099(23)00372-9*—This Article should have been published under a CC BY Gold Open Access license. This correction has been made to the online version as of Sept 27, 2023, and will be made to the printed version.

