# Supplementary material for: Burden of tuberculosis among vulnerable populations worldwide: an overview of systematic reviews
Source: Lancet Infect Dis. 2023 Dec;23(12):1395–407. doi: 10.1016/S1473-3099(23)00372-9 (PMC10665202; doi:10.1016/S1473-3099(23)00372-9)
Supplement: Supplementary appendix [file mmc1.pdf]

THE LANCET
Infectious Diseases

Supplementary appendix

This appendix formed part of the original submission and has been peer reviewed. We post it as supplied by the authors.

Supplement to: Litvinjenko S, Magwood O, Wu S, Wei X. Burden of tuberculosis among vulnerable populations worldwide: an overview of systematic reviews. *Lancet Infect Dis* 2023; published online Sept 8. https://doi.org/10.1016/S1473-3099(23)00372-9.

Burden of tuberculosis among vulnerable populations worldwide: an overview of systematic reviews

Supplementary Appendix

Table of Contents

1. Medline Search Strategy.......... 2

2. Inclusion and Exclusion Criteria.......... 4

3. Full-Text Screening List of Exclusion Reasons.......... 5

4. Supplementary Table 1: Characteristics of Included Studies and Summary of Most Recent Data on TB Burden in Vulnerable Populations (Complete Results).......... 6

5. Supplementary Figure 1: Forest Plot of TB Prevalence Estimates Among Vulnerable Populations.......... 20

6. Supplementary Figure 2: Forest Plot of TB Prevalence Estimates Among PLHIV.......... 22

7. Supplementary Figure 3: Forest Plot of TB Incidence Estimates Among Vulnerable Populations.......... 23

8. Supplementary Maps: Availability of Studies Reporting Data on the Prevalence and/or Incidence of TB Disease in Vulnerable Population Groups between 2010 and 2023, by Country and Background TB Burden 24

9A. AMSTAR 2 Assessments of Included Reviews.......... 29

9B. Modified JBI Critical Appraisal Checklist for Primary/Database Studies 31

1. Medline Search Strategy

MEDLINE: Epub Ahead of Print, In-Process & Other Non-Indexed Citations, Ovid MEDLINE® Daily and Ovid MEDLINE® <1946 to March 9, 2023>

1	*tuberculosis/ or *latent tuberculosis/ or *tuberculosis, pleural/ or *tuberculosis, pulmonary/ or *Mycobacterium tuberculosis/	Tuberculosis terms - focus
2	(vulnerable or vulnerability).ti,ab,kf.	
3	(definition* or define* or criteria* or "key population" or "high priority" or "high risk" or "at risk" or "disproportionate*" or framework* or dynamic* or susceptibl* or factor or factors).ti,ab,kf.	
4	risk/ or risk factors/ or morbidity/ or incidence/ or prevalence/	
5	or/2-4	Definition or risk terms
6	Vulnerable Populations/ [****MeSH since 2003****]	
7	("Foreign-born" or "foreign born").ti,ab,kf.	
8	disabled persons/ or disabled children/ or persons with mental disabilities/ or mentally ill persons/ or disaster victims/ or "emigrants and immigrants"/ or undocumented immigrants/ or enslaved persons/ or homeless persons/ or homeless youth/ or medically uninsured/ or prisoners/ or refugees/ or sex workers/ or "sexual and gender minorities"/ or intersex persons/ or transgender persons/ or "transients and migrants"/ or veterans/ [****Misc. marginalization terms****]	
9	(displaced adj2 (people* or person* or population* or internally)).ti,ab,kf.	
10	continental population groups/ or african continental ancestry group/ or african americans/ or american native continental ancestry group/ or indians, central american/ or indians, north american/ or alaskan natives/ or indigenous canadians/ or inuits/ or american natives/ or indians, south american/ or asian continental ancestry group/ or asian americans/ or european continental ancestry group/ or oceanic ancestry group/ or ethnic groups/ or amish/ or arabs/ or indigenous peoples/ or roma/ or hispanic americans/ or mexican americans/ or jews/	
11	(indigenous or aboriginal* or nomad* or (mobile adj2 population*)).ti,ab,kf.	
12	substance-related disorders/ or alcohol-related disorders/ or alcohol-induced disorders/ or exp alcohol-induced disorders, nervous system/ or cardiomyopathy, alcoholic/ or fetal alcohol spectrum disorders/ or exp liver diseases, alcoholic/ or pancreatitis, alcoholic/ or psychoses, alcoholic/ or alcoholic intoxication/ or alcoholism/ or binge drinking/ or amphetamine-related disorders/ or cocaine-related disorders/ or exp drug overdose/ or inhalant abuse/ or marijuana abuse/ or neonatal abstinence syndrome/ or phencyclidine abuse/ or psychoses, substance-induced/ or substance abuse, intravenous/ or substance abuse, oral/ or substance withdrawal syndrome/ or alcohol withdrawal delirium/ or alcohol withdrawal seizures/ or "tobacco use disorder"/ or drug user/	
13	smoking/ or pipe smoking/ or water pipe smoking/ or smoking, non-tobacco products/ or cocaine smoking/ or marijuana smoking/ or tobacco smoking/ or cigar smoking/ or cigarette smoking/ or vaping/	
14	narcotic-related disorders/ or opioid-related disorders/ or heroin dependence/ or morphine dependence/ or opiate overdose/ or opium dependence/ or substance abuse, oral/ or substance withdrawal syndrome/ or alcohol withdrawal delirium/ or alcohol withdrawal seizures/ or "tobacco use disorder"/	
15	(pwud or pwid).ti,ab,kf.	
16	socioeconomic factors/ or economic factors/ or economic status/ or educational status/ or academic failure/ or literacy/ or employment/ or career mobility/ or child labor/ or employment, supported/ or personnel downsizing/ or return to work/ or teleworking/ or unemployment/ or workplace/	
17	income/ or pensions/ or employee retirement income security act/ or remuneration/ or "salaries and fringe benefits"/ or family leave/ or parental leave/ or health benefit plans, employee/ or sick leave/	
18	medical indigency/ or poverty/ or poverty areas/ or social change/ or social class/ or social mobility/ or social conditions/	
19	residence characteristics/ or catchment area, health/ or housing/ or housing for the elderly/ or public housing/ or independent living/	
20	rural health/ or suburban health/ or urban health/	
21	((working or urban or rural) adj2 (poor or poverty)).ti,ab,kf.	
22	(slum adj2 dweller*).ti,ab,kf.	
23	overcrowding.ti,ab,kf.	
24	family characteristics/ or marital status/ or divorce/ or marriage/ or single person/ or single parent/ or widowhood/	
25	(household* adj2 contact*).ti,ab,kf.	
26	health education/ or health literacy/ or health fairs/	
27	dehumanization/ or commodification/ or social adjustment/ or social conformity/ or social isolation/ or social marginalization/ or social stigma/ or stereotyping/	
28	attitude to health/ or health knowledge, attitudes, practice/ or "treatment adherence and compliance"/ or "patient acceptance of health care"/ or patient compliance/ or medication adherence/ or directly observed therapy/ or no-show patients/ or patient dropouts/ or patient participation/ or patient satisfaction/ or patient preference/ or treatment refusal/ or vaccination refusal/	
29	"Social Determinants of Health"/	
30	(social adj2 determinant* adj2 (TB or tuberculosis)).ti,ab,kf.	
31	minors/ or farmers/ or metal workers/ or miners/	
32	(mines or miners or ((tea or coffee or sugar or plantation*) adj2 work*) or farmer* or ((industrial or factory or brick or cement or quarry or garment*) adj2 worker*)).ti,ab,kf.	
33	occupational exposure/ or maximum allowable concentration/ or "threshold limit values"/ or war exposure/	

34	malnutrition/ or deficiency diseases/ or avitaminosis/ or ascorbic acid deficiency/ or scurvy/ or vitamin a deficiency/ or vitamin b deficiency/ or choline deficiency/ or folic acid deficiency/ or hyperhomocysteinemia/ or pellagra/ or riboflavin deficiency/ or thiamine deficiency/ or beriberi/ or wernicke encephalopathy/ or vitamin b 6 deficiency/ or vitamin b 12 deficiency/ or anemia, pernicious/ or subacute combined degeneration/ or vitamin d deficiency/ or rickets/ or osteomalacia/ or "chronic kidney disease-mineral and bone disorder"/ or rickets, hypophosphatemic/ or familial hypophosphatemic rickets/ or vitamin e deficiency/ or steatitis/ or vitamin k deficiency/ or vitamin k deficiency bleeding/ or magnesium deficiency/ or potassium deficiency/ or protein deficiency/ or protein-energy malnutrition/ or swayback/ or severe acute malnutrition/ or kwashiorkor/ or starvation/	
35	malnourish*.ti,ab,kf.	
36	hiv infections/ or acquired immunodeficiency syndrome/ or acute retroviral syndrome/ or aids arteritis, central nervous system/ or aids dementia complex/ or aids-associated nephropathy/ or aids-related complex/ or aids-related opportunistic infections/ or hiv enteropathy/ or hiv seropositivity/ or hiv wasting syndrome/ or hiv-associated lipodystrophy syndrome/ or HIV Seronegativity/	
37	plhiv.ti,ab,kf.	
38	diabetes mellitus/ or diabetes mellitus, type 1/ or wolfram syndrome/ or diabetes mellitus, type 2/ or diabetes mellitus, lipoatrophic/ or diabetes, gestational/ or diabetic ketoacidosis/ or donohue syndrome/ or latent autoimmune diabetes in adults/ or prediabetic state/	
39	Pregnant Women/ or postpartum period/ or lactation/ or milk ejection/ or pregnancy rate/ or pregnancy trimesters/ or pregnancy trimester, first/ or pregnancy trimester, second/ or pregnancy trimester, third/ or exp pregnancy/ or pregnancy, complicaitons, infectious/ or climacteric/ or andropause/ or menopause/ or menopause, premature/ or perimenopause/ or postmenopause/ or premenopause/	
40	(*tuberculosis/ or *latent tuberculosis/ or *tuberculosis, pleural/ or *tuberculosis, pulmonary/) and treatment outcome/ [****Indexing for post TB sequelae****]	
41	("post Tuberculosis sequela*" or "post-tuberculosis sequela*").ti,ab,kf.	
42	or/6-41	Vulnerable population terms
43	1 and 5 and 42	
44	limit 43 to (english or French or Chinese)	
45	limit 44 to yr="2010 -Current"	

Websites of major public health agencies and NGOs searched for relevant grey literature using key words including “vulnerable”; “vulnerability”; “TB vulnerable populations”; “TB key populations”, TB at-risk populations”

- US Centers for Disease Control and Prevention (CDC)
- European Centers for Disease & Control (ECDC)
- Public Health England (PHE)
- Africa Centers for Disease Control and Prevention (Africa CDC)
- Public Health Agency of Canada (PHAC)
- World Health Organization (WHO)
- The Global Fund
- Stop TB Partnership
- United States Agency for International Development (USAID).

2. Inclusion and Exclusion Criteria

<table>
<tr><th></th><th>Inclusion criteria</th><th>Exclusion criteria</th></tr>
<tr><td>Population</td><td>Populations vulnerable to tuberculosis (as defined by our conceptual framework), experiencing concurrent risk of TB disease, disadvantaged socioeconomic status, and limited access to health systems.

Key populations of interest include:
1. People experiencing homelessness
2. Incarcerated populations
3. Indigenous people
4. People living in slum settings
5. Refugees, asylum seekers, internally displaced persons (IDPs), and undocumented migrants
6. Miners
7. Nomadic populations
8. Female sex workers (FSW)
9. Men who have sex with men (MSM)
10. Transgender individuals
11. People who use drugs (PWUD)
12. People living with HIV (PLHIV)</td><td>Populations with increased susceptibility to tuberculosis on the basis of their biological vulnerability (susceptibility) to TB disease without other forms of social exclusion.

Populations which are ineligible for inclusion:
1. General migrants and immigrants (e.g., economic migrants, international students, foreign born individuals)
2. People living with diabetes and other chronic illnesses, patients suffering from silicosis, receiving dialysis, undergoing organ transplantation etc.
3. Healthcare workers
4. Pregnant individuals
5. Children and the elderly
6. Household and other contacts</td></tr>
<tr><td>Condition</td><td>TB disease:
Active tuberculosis disease includes all forms of pulmonary and extra-pulmonary tuberculosis. A definitive diagnosis of active TB disease is made using microscopy and culture or molecular tests or presumptive diagnosis using clinical or imaging techniques.</td><td>Studies whose primary focus is genetic or molecular epidemiology of TB, LTBI, M/XDR-TB, extrapulmonary TB, bovine TB/ zoonotic related transmission, publications evaluating diagnostic test sensitivities and performance, treatment outcomes (i.e., adherence, survival, implementation science), and studies on knowledge, attitude, and practices.

All other infectious diseases and comorbidities, (with the exception of HIV co-infection)</td></tr>
<tr><td>Context</td><td>Global, regional, or country-level empirical evidence. We included publications which reported point prevalence or period prevalence as a proportion, percentage (%) or as the number of cases per 100,000 people. We included publications which reported cumulative incidence (as a proportion) or incidence rate (in person-years). Secondary outcomes of interest included comparisons with general populations (i.e. prevalence ratio (PR) or incidence ratio (IR)).</td><td>Reviews concerning animal studies/models, vaccine trials (R&D) and efficacy, health economics / TB expenditure ($), mathematical modeling works, outbreak investigations, and studies with an overly specific or small study population not considered generalizable such as localities, or single-centre, institutional settings.</td></tr>
<tr><td>Study designs</td><td>Systematic or scoping reviews published in English, French or Chinese from 2010-2023.

If none or few reviews were identified, we considered primary prevalence studies.</td><td>Reviews published prior to 2010; reviews published in other languages.</td></tr>
</table>

3. Full-Text Screening List of Exclusion Reasons

1. Full text unavailable:
Full-text was not retrieved through available institutional privileges, and in instances of direct request through author correspondence was still not retrieved.

2. Wrong outcome (systematic reviews):
Studies addressing the correct target populations, however outcome measures are not related to active TB burden and/or risk. Examples include the assessment of TB symptoms, treatment, service models, or knowledge, attitude and practices.

3. Wrong population (systematic reviews):
Upon full-text review, the study population is not as expected from title/abstract screening, is not a vulnerable group, or TB is not considered a main point.

5. Publication type (systematic reviews):
In relation to systematic reviews, a global or regional systematic review or meta-analysis has been identified on the same or similar topic, and/or data is outdated, lending more localized reviews to be less generalizable.

6. Primary study or SDA (not required):
This refers to a wide variety of articles deemed valid to study objectives, but are considered of lower scope or quality as a systematic review and/or meta-analysis on the group in focus has already been identified. The selection of higher quality studies is meant to convey that this publication domain is saturated, and therefore smaller-scale publications are considered less generalizable. This category can also refer to primary studies which are already included under selected systematic reviews, to avoid double-citation.

7. Primary study or SDA (not appropriate):
This refers to primary literature which has not met the inclusion/exclusion criteria based on any of: wrong outcomes, population or study design.

8. Other secondary literature:
Narrative reviews; any publication of secondary data that does not follow established methods for systematic or scoping reviews (i.e. systematic search and/or inclusion/exclusion criteria). May include commentaries or other works with correct populations and/or subject matter but data is not available, extractable or fit for purpose.

9. Other reasons:
Any reason as not mentioned. This can include protocols, author correspondence or letters to the editor etc.

SDA=secondary analysis (database) studies

4. Supplementary Table 1: Characteristics of Included Studies and Summary of Most Recent Data on TB Burden in Vulnerable Populations (Complete Results)

Author (year), Period of study	Type of study, setting	Number of included studies (for reviews), Sample Size	Prevalence per 100,000 or percentage, (confidence interval 95%); # of studies (for reviews)	Comparison with general population† [PR], (confidence interval 95%)	Incidence per 100,000 person-years or percentage, (confidence interval 95%); # of studies (for reviews)	Comparison with general population† [IRR], (confidence interval 95%)	Subgroup analyses and/or data available
People experiencing homelessness							
Beijer (2012)<br><br>1980-2012	Review & MA, Global<br><br>Combined TB, HCV, and HIV literature<br><br>(n= 8 countries) [TB prevalence]	17 [TB specific studies], n= 43,605	Pooled estimate: 1·1% (0·8–1·5); 17<br><br>Range of prevalence: 0·2-7·7%	Range 22·4 (Sweden) to 461 (USA); 17	NR	NR	meta-regression:<br>- Sex<br>- Country<br>-*Diagnostic method<br>- Sample size<br>- Year of publication<br>- *Population prevalence
Gioseffi (2022)<br><br>2014-2020	Review, Global<br><br>(n= 7 countries)	7, n= 98,810	- Ethiopia: 2·6%<br>- USA: 4·1%	NR	- Ethiopia: 505<br>- Portugal: 122<br>- India: 270	NR	-TB-HIV co-infection<br>- Presence of vulnerability factors (individual, social, or programmatic)
Hino (2021)<br><br>Up to 2018	Review, Global<br><br>(n= 3 countries)	5, NR	Values are for homeless among all notified cases (general population inclusive)<br>- Japan: 1·4%<br>- Brazil: 2·8%<br>- USA (1994-2003): 6·4%<br>- USA (1994-2010): 6%	NR	- USA (1994-2010): 36-47 (for data from 2006-2010)<br>- USA (1995-2004): 411	- USA (1994-2010): 10<br>- USA (1995-2004): 43·5	-TB-HIV co-infection<br>- Diabetes<br>- Alcohol, drug use
Self (2021)<br><br>2007-2016	Secondary data analysis (national surveillance), USA	NR	NR	NR	- 46 in 2007 to 31 in 2016, rates are plotted annually. National average of 36, 2011- 2016	- 10·7 based on 2016 national rate, rates are plotted annually	- Incidence rates for 21 urban areas with PEH population ≥10,000;
Incarcerated populations							
Cords (2021)<br><br>1980-2020	Review & MA, Global<br><br>Unspecified # of countries	106, n= 6,727,513 [prevalence]<br><br>51, n=1,858,323 [incidence]	Pooled Estimates:<br><br>- North America: 320 (130-650); 12	NR	Pooled Estimates:<br><br>- North America: 30 (20-50); 12	Overall: 10·1 (7·6–13·0); 47<br><br>- North America: 4·1 (2·8–6·2); 7	- High burden country classification<br>- National incidence<br>- Surveillance method<br>- Study design

			- South America:<br>1,680 (830-2,970); 16<br>- European:<br>1,000 (510-1,770); 18<br>- African:<br>1,610 (980-2,500); 30<br>- South-East Asia:<br>1,810 (670-4,000); 8<br>- Western Pacific:<br>720 (270-1,600); 10<br>- Eastern Mediterranean:<br>1,160 (480-2,370); 11		- South America:<br>970 (460-1,860); 12<br>- European:<br>610 (310-1,100); 11<br>- African:<br>2,190 (810-4,840); 7<br>- South-East Asia:<br>1,550 (240-5,300); 3<br>- Western Pacific:<br>390 (80-1,130); 3<br>- Eastern Mediterranean:<br>270 (50-880); 3	- South America:<br>26·9 (17·1–40·1); 12<br>- European:<br>8·7 (3·7–16·8); 6<br>- African:<br>12·6 (6·2–22·3); 6<br>- South-East Asia:<br>11·7 (4·1–27·1); 3<br>- Western Pacific:<br>6·8 (2·9–13·2); 3<br>- Eastern Mediterranean:<br>15·6 (6·5–32·5); 3	- Setting (prison, jail, or detention centre)<br>- Study years of data collection<br>- National income<br>- TB-HIV co-infection<br>- Injection drug use
Dolan (2016)<br><br>2005-2015	Review & MA, Global<br><br>Combined TB, HCV, HBV, HIV literature<br><br>(n= 27 countries) [TB prevalence]	299 [all studies combined], NR	<u>Pooled Estimates:</u><br>Overall:<br>2,790 (2,050-3,650)<br>- East and Southern Africa:<br>5,330 (2,066-9,998)<br>- West and Central Africa:<br>2,946 (2,362-3,591)<br>- Asia and Pacific:<br>1,173 (712-1,747)<br>- Eastern Europe & Central Asia: 4,903 (1,846-9,321)<br>- Latin America:<br>1,977 (1,347-2,726)<br><br><u>Data not pooled for:</u><br>- Middle East & North Africa; 1, North America; 1, Western Europe;2, Caribbean: (no data)	NR	Visually represented (n= 14 countries)	NR	- Sex<br>- TB-HIV co-infection<br>- PWID<br>- FSWs, MSM, transgender people (no data)
Baussano (2010)<br><br>1980-2010	Review, Global<br><br>(n= 8 countries)	19, n= at least 512,780 (6 studies not reporting)	<u>NR</u>	NR	High-income countries: median 238 (IQR 156-639)<br>Range of incidence:<br>- Spain: 639-6,799; 4<br>- Israel: 25<br>- Hong Kong: 260<br>- USA: 61-274; 6<br>- France: 216<br><br>Middle/low-income countries:<br>median 1,943<br>(IQR 1,045-2,778)<br>Range of incidence:<br>- Brazil: 1,045-2778; 2<br>- Russia: 880-2,035; 3	Median 23·0<br>(IQR 11·7-36·1)<br><br>High-income countries:<br>17·9 (IQR 8·6-61), range 2·5 (1·7 -3·8) (Israel) to<br>151 (134 -170) (Spain)<br><br>Middle/low-income countries:<br>32·8 (IQR 15·4-36·1), range 8·8 (5·8 -13·4) (Russia) to 39·1 (Russia)	- Estimated fraction of TB in the general population attributable to prison transmission<br>- Incarcerated population per 1,000 inhabitants

					- Ivory Coast: 5,803		
Kinner (2018)* *age-stratified re-analysis of Dolan 2016 data	Review, Global<br><br>Combined TB, HCV, HBV, HIV literature<br><br>(n= 9 countries) [TB prevalence]	11 [TB specific studies], NR	Adolescents and young adults (AYA) (<25 years)<br>- Cameroon 3·7% (2·6-5·2)<br>- Bangladesh 1·1% (0·6-1·8)<br>- Two studies (Iran, US) identified no young prisoners with active TB (i·e 0·0%)<br><br>Older prisoners (>25 years)<br>- Range 0·7% (0·1-2·1) Brazil to 5·0% (3·7-6·6) Tajikistan; 10<br><br>Mixed age groups (e.g., age 20–30 years)<br>- Range: 0·4% (0·0-1·3) Iran to 3·7% (2·0-6·3) South Africa; 9				- *AYA prisoners vs. older prisoners<br>- AYA sex stratified estimates (no data)
Placeres (2023)<br><br>Up to 2021	Review & MA, Global<br><br>(n= 31 countries)	59, n= 1,012,448	Pooled estimate:<br>2·6% (2·1-3·3); 44<br><br>Pooled estimate by high-burden countries:<br>3·5% (2·7-4·6); 29<br>Non high-burden countries:<br>1·4% (0·9-2·4); 15	NR	NR	NR	- Country income level<br>- Trend in prevalence<br>- Study quality
Dianatinasab (2018)<br><br>Up to 2017	Review & MA, Global<br><br>TB in HIV+ prisoners<br><br>(n=11 countries)	22, n= 220,101 total prisoner population	1,611 cases of TB among 6,126 HIV-positive prisoners<br><br>Pooled estimate:<br>32·6% (27·5-38·2)<br><br>Pooled estimate, by region:<br>- Africa: 14% (8-24); 6<br>- Europe: 25% (12-45); 6<br>- Asia: 35% (12-68); 6<br>- North/South America: 37% (31-44); 4<br><br>Range of prevalence:<br>6% (Zambia, Iran, South Africa) to 94% (Iran)	NR	NR	NR	- Duration of incarceration<br>- Cell congestion<br>- History of previous incarceration<br>- Diagnostic method<br><br>meta-regression;<br>- Year of study

Edge (2016)<br><br>Up to 2015	Review,<br>Global<br><br>TB/HIV co-infection (TB in HIV+, HIV in TB+)<br><br>(n= 20 countries)	41 [37 prevalence, 4 incidence], NR	Co-infection, range of prevalence:<br>0·4% (Brazil) to 6·7% (Spain); 3<br><br>TB in HIV+, range of prevalence:<br>6·4% (Zambia) to 63% (Spain); 8<br><br>HIV in TB+, range of prevalence:<br>0·0% (Brazil) to 73% (Ethiopia); 33	- Iran: 1·8<br>- Malaysia: 165<br>- Spain: 1·9<br>- Ukraine: 1·0<br>- Russia: 7·2	- USA: 359 (214-725) in HIV+ inmates compared to 20 (14-28) in HIV- inmates<br>- Nigeria: 24% HIV+ inmates with positive TST developed active TB compared to 13·8% in HIV- inmates<br>- Mexico: 7,700	- Spain: Relative risk of TB in HIV+ prisoners 4·1 (2·6-6·4)	- Measures of association between TB and HIV positivity<br>- History of incarceration<br>- Setting
Kuupiel (2020)<br><br>2000-2019	Review, sub-Saharan Africa<br><br>(n= 11 countries)	31, NR	Range of prevalence:<br>- Ethiopia: 3·4-21·9%; 13<br>- Nigeria: 1·2-21·2%; 3<br>- Zambia: 4·2-7·6%; 4<br>- Democratic Republic of the Congo (DRC): 17·7-21·7%; 2<br>- Uganda: 2·0-2·3%; 2<br>- South Africa: 2·7-3·5%; 2<br>- Malawi: 0·7%<br>- Burkina Faso: 1·3%<br>- Ghana: 0·7%<br>- Cameroon: 3·5%<br>- Côte d'Ivoire: 6·2%	NR	NR	NR	- Type of ACF approach<br>- Diagnostic method
Poteat (2018)<br><br>Up to 2017	Review,<br>Global<br><br>Combined HIV, STI, VH literature<br><br>(n= 2 countries) [TB prevalence]<br><br>Incarcerated transgender people	2 [TB specific studies], n= 199	- Sao Paolo, Brazil, 1990: 13% of 82 incarcerated transgender people reported a history of TB<br>- Milan, Italy, 2013: 10·3% of 117 incarcerated transgender people had positive TB screening results	- (Milan)<br>OR 4·9 (2·1 -11·7) compared to incarcerated cisgender persons			[no data available]<br><br>- Access to gender-affirming care<br>- Housing segregation<br>- Physical & sexual violence
Dadu (2021)<br><br>2014-2018	Secondary data analysis (national surveillance), WHO European Region<br>(n= 53 countries)	NR	NR	NR	- 42/53 (79%) countries reporting incident TB cases (new and relapse) in the civilian and penitentiary sectors	- Incident TB rates: 4-24<br>- Relative risk of developing active TB: ranged from 3·0 (2·0-4·6) U.K. to 57 (35-92) Slovakia	- Trends in notification rate<br>- Subtotals available by EECA/ non-EECA region

Indigenous people							
Tollefson (2013)<br><br>1990-2011	Review, Global<br><br>(n= 19 countries)	91, NR	- African:<br>1,800 (Peul/Dogon peoples; Mali) and 4,600 (nomadic Fulani; Chad); 2<br>- North America: NR<br>- Latin America and Caribbean: Range 211 (Xavante, Brazil) to 6,700 (Indigenous, Ecuador); 8<br>- Eastern Mediterranean: NR<br>- European:<br>Range 85 (Inuit, Greenland) to 1,160 (Chukotka Autonomous Indigenous, Russia); 4<br>- South-East Asian:<br>Range 0 (Forest peoples, India) to 46,197 (Saharia, India); 17<br>- Western Pacific:<br>47 (Bai, China) and 395 (Ethnic minorities, not specified, China); 2	Comparator population‡<br>- African:<br>Range 5·5 (Peul/Dogon peoples; Mali) to 10·7 (nomadic Fulani; Chad); 2<br>- North America: NR<br>- Latin America and Caribbean:<br>Range 3·8 (Xavante, Brazil) to 94 (Yanomami, Brazil-Amazonas); 8<br>- Eastern Mediterranean: NR<br>- European:<br>Range 0·9 (Indigenous, Russia) to 15·3 (Inuit, Greenland); 4<br>- South-East Asian:<br>Range 0·5 (Baiga & Jawadhu-Hills tribes, India) to 138 (Saharia, India); 17<br>- Western Pacific:<br>0·4 (Bai, China) to 3·1 (Ethnic minorities, not specified, China); 2	- African: NR<br>- North America:<br>Range 4·3 (American Indian/Alaska Native; USA) to 431 (Indigenous; Canada); 23<br>- Latin America and Caribbean: Range 42·3 (Amazonic Aguaruna, Peru) to 3,700 (Ache natives, Paraguay); 11<br>- Eastern Mediterranean:<br>18·1 (Bedouins, Israel); 1<br>- European:<br>Range 85 to 304 (Chukotka Autonomous Indigenous, Russia); 4<br>- South East Asian:<br>Range 770 (Tibetan refugees, India) to 4,571 (Tibetan refugees, Canada); 5<br>- Western Pacific:<br>Range 0 (Aborigines/Torres Strait Islanders descendants, Australia) to 6,500 (E-Lun Chun (Ortochen), China); 16	Comparator population‡<br>- African: NR<br>- North America:<br>Range 0·3 (Yukon, Canada) to 331 (Saskatchewan, Canada); 23<br>- Latin America and Caribbean:<br>Range 0·4 (Shapra, Peru) to >1,000 (Indigenous, Brazil); 11<br>- Eastern Mediterranean:<br>2·1 (Bedouins, Israel); 1<br>- European:<br>Range 2·3 (Chukotka Autonomous Indigenous, Russia) to 10 (Indigenous, Russia); 4<br>- South East Asian:<br>Range 3·6 (Tibetan refugees, India) to 762 (Tibetan refugees, USA); 4<br>- Western Pacific:<br>Range 0·6 (Aborigines/Torres Strait Islanders descendants, Australia) to 42·5 (E-Lun Chun (Ortochen), China); 16	- Data granularity (any of national, province/ state/region, city, district, village/ town/reserve)

Gilmour (2022)<br><br>Up to 2020	Review & MA, South-East Asia and the Western Pacific Region<br><br>(n= 4 countries)<br><br>Minority Indigenous populations	24, n= 337,677	Pooled estimate:<br>2·3% (1·7–2·9); 24<br><br>Range of prevalence:<br>0·3% (0·2–0·4) to<br>32·0% (24·6–40·5)<br><br>Pooled estimate, by region:<br>- South-East Asia:<br>2·2% (1·6–3·0); 18<br>- Western Pacific:<br>2·3% (0·7–4·9); 6	No difference in prevalence between minority Indigenous populations<br>5·0%<br>(1·7–9·9); 4<br>and non 'minority indigenous' populations<br>5·0%<br>(0·3–14·2); 4	NR	NR	- Minority Indigenous population groups<br>- Age<br><br>meta-regression:<br>- WHO Region<br>- WHO mortality strata<br>- Country of study<br>- Year of data collection<br>- Sex<br>- Diagnostic/ screening method
Ferreira (2020)<br><br>2011-2017	Secondary data analysis (national surveillance), Brazil	NR	NR	NR	109 (mean incidence), lowest in 2014 at 102, highest in 2011 at 120<br><br>(6,520 cases in Indigenous people between 2011-2017)	NR	- Regional analysis (national, regional, & federative units)<br>- Trends in notification rate
Springer (2021)<br><br>2009-2019	Secondary data analysis (national surveillance), USA<br><br>American Indian or Alaskan Native (AIAN) and Native Hawaiian or other Pacific Islander (NHPI)	NR	NR	NR	- AIAN: range 7·5 in 2010 to 3·7 in 2019<br>- NHPI: range 1·2 in 2013 to 8·4 in 2017	[Comparator 'white' general population]<br>- AIAN:<br>range 6·5 (5·3–8·0) in 2009 to 13·5 (11·3–16·1) in 2015<br>- NHPI:<br>range 2·1 (0·5–4·4) in 2013 to 20·1 (13·9–28·9) in 2017	- Age-adjusted incidence rates<br>- Age-adjusted IRR<br>- Clinical and sociobehavioural risk factors (e.g., region, sex, *history of TB, *positive sputum, *disease site, *renal disease, *diabetes, HIV+, immunosuppressed, *recent transmission, *alcohol/drug use, *homelessness, *occupation, *incarcerated)
Thomas (2021)<br><br>2015-2020	Cross-sectional, 17 states, India<br><br>Tribal populations	n= 74,532	432 (373-491)	NR	NR	NR	- Diagnostic method<br>- State<br>- Zone<br>- *Age group<br>- *Male gender<br>- Occupation<br>- *Alcohol/smoking<br>- *Treatment history<br>- *Low BMI
People living in slum settings							

Noykhovich (2019)<br><br>1993-2017	Review & MA, Global<br><br>(n= 13 countries)	22<br><br>3, n= 7,409 [prevalence]<br><br>19, n= 2,406,397 [incidence]	- Pakistan: 329<br>- Uganda: 3,548<br>- Philippines: 670	Range 1·6 (Philippines) to 5·5 (Uganda)	Range of incidence:<br>- Bangladesh: 192<br>- Brazil: 72-1,365; 3<br>- Cambodia: 233<br>- Haiti: 388-1,165; 2<br>- India: 5·1-4,219; 3<br>- Iran: 17·5<br>- Nepal: 508<br>- Nigeria: 4,163<br>- Pakistan: 499-1,886; 2<br>- Peru: 8,825<br>- South Africa: 617-1,575; 2<br>- Uganda: 370	Range<br>0·02 (India) to 58 (Peru)<br><br>Pooled OR compared to national rates<br>3.0 (2.8-3.1); 22	-Diagnostic method<br>- Type of case finding<br>- TB-HIV co-infection
**Refugees, asylum seekers, internally displaced persons (IDPs), undocumented migrants**							
Arshad (2010)<br><br>Up to 2008	Review & MA, Global<br><br>(n= 13 host countries)<br><br>Refugees, asylum seekers, immigrants	22, n= 2,620,739<br><br>Refugees<br>n= 18,982<br><br>Asylum seekers<br>n= 135,265<br><br>Regular immigrants<br>n= 2,466,492	Pooled estimates:<br>- Refugees:<br>1,192 (678–1,717); 7<br>- Asylum seekers:<br>270 (198–342); 7<br>- Regular immigrants:<br>284 (204–364); 7<br>- Overall: 349 (290–408)<br><br>Range of prevalence:<br>101 (regular immigrants) to 3,811 (refugees)	Compared to autochthonous population:<br>- Refugees:<br>131 (59–290); 7<br>- Asylum seekers:<br>30·1 (19·3–47·1); 7<br>- Regular immigrants:<br>29·4 (9·7–89); 7<br><br>- Overall:<br>48·2 (23·3–100)	NR	NR	- Routine screening programs only<br>- Geographic origin (world region)<br>- Only studies from European countries<br>- Sex
Bozorgmehr (2017)<br><br>Up to 2016	Review & MA, Germany<br><br>Asylum seekers	6, n= 89,294	Pooled estimate:<br>347 (178-573)<br><br>Range of prevalence:<br>72 (45-110) to<br>641 (419-937)<br><br>Pooled overall yield combining asylee data from Arshad 2010:<br>304 (224–367); 13	NR	NR	NR	- Countries of origin<br>- Sampling strategy<br>- Diagnostic method<br>- Sex
Proenca (2020)<br><br>2000-2017	Review & MA, Global<br><br>Unspecified # of countries<br><br>Refugees, asylum	46, n= 537,218<br><br>Refugees<br>n=437,264<br><br>Asylum seekers<br>n= 95,283	Pooled estimate by country of origin:<br>1,331 (542-2,384); 16<br><br>Pooled estimate by host continent:<br>-Americas: 1,080 (405-	NR	NR	NR	NR

	seekers	Mixed, n= 4,671	2,035); 3<br>- Asia: 860 (0-3,588); 3<br>- Europe: 1,458 (570-2,648); 12<br><br>Range of prevalence:<br>0 (USA, Australia, refugee) to 11,364 (Syria, refugee)				
Meaza (2022)<br><br>2014-2021	Review,<br>Global<br><br>Refugee and migrant populations	6, n= 2,115,223<br><br>Immigrants and refugees<br>n= 1,784,685<br><br>Refugees<br>n= 38,403<br><br>Asylum seekers<br>n= 41,196	Asylum seekers: 93 (Friedland, Germany)<br><br>Asylum seekers: 535 (Bologna, Italy)<br><br>Refugees: 19 (Turkey)	Asylum seekers: 17.5 (Germany)<br><br>Asylum seekers: 80 (Italy)	Migrants and refugees: 19 (Alberta, Canada)<br><br>Immigrants and refugees: 258 (USA)<br><br>Refugees: 431 (India)<br><br>Asylum seekers: 754 (Netherlands)	Migrants and refugees: 4·0 (Alberta)<br><br>Refugees: 2·4 (India)<br><br>Asylum seekers: 164 (Netherlands)	- Countries of origin
Abdullahi (2020)<br><br>2017-2018	Controlled intervention study, northeast Nigeria<br><br>Internally displaced persons	n= 283,556 screening encounters in IDPs<br><br>By site:<br>- IDP camps: 90,738<br>- Host communities: 192,818	- IDP camps:<br>598 per 100,000 screening encounters<br>- IDPs Host Communities: 428 per 100,000 screening encounters<br><br>- Overall 502 per 100,000 screening encounters	- 10x national notification rates, and 2x the estimated national incidence	NR	NR	- TB-HIV co-infection<br>- Post-intervention trend differences in notification rates between intervention and control areas
Miners							
Rambiki (2020)<br><br>2019	Cross-sectional, Malawi<br>(multiple districts)<br><br>Active miners in the formal and informal sector	n= 2,013	13,512	40	NR	NR	- TB-HIV co-infection<br>- *District<br>- Sex<br>- *Age<br>- *Educational level<br>- *Marital status<br>- *Informal vs. formal mining<br>- *Mining activities<br>- *Years of mining
Ngosa (2016)<br><br>2005-2010	Secondary data analysis, (cross-sectional), Zambia	n= 357	9,524	24	NR	NR	- *Age at certification<br>- *Smoking status<br>- Sex<br>- *Length of service

	Active copper miners						- *Medium and high cumulative respirable silica dust exposure categories
Moyo (2022)<br><br>2020-2022	Secondary data analysis, (cross-sectional), Matabeleland South and Midlands provinces, Zimbabwe<br><br>Artisanal and small-scale miners	n= 3,821	6·7% (6·0-7·6)	NR	NR	NR	- *Sex<br>- *Age<br>- *Education<br>- *HIV status<br>- *Silica exposure<br>- *History of TB<br>- *Pneumonia<br>- Asthma<br>- *Alcohol intake<br>- *Duration as miner<br>- *Type of facility
Ohene (2021)<br><br>2017-2018	Secondary data analysis, (national surveillance), southwest Ghana<br><br>Informal gold mining communities	n= 10,441 community members<br><br>(miners made up 5-10% of each sampled region)	2,725	7.7	NR	NR	- *Age<br>- *Sex<br>- *Occupation<br>- *Region<br>- *Symptoms
Nomadic populations							
John (2015)<br><br>2012-2013	Cross-sectional, Adamawa state, Nigeria	n= 96,376	1,359	4·2	NR	NR	- Sex<br>- TB-HIV co-infection<br>- Historical and intervention case notification rates
Honarvar (2014)<br><br>2012-2013	Cross-sectional, Fars province, Iran	n= 5,506	73	9	NR	NR	- Number of persons, families, tents<br>- Tent population<br>- Age<br>- Sex<br>- Education
Lo (2016)<br>2012	Cross-sectional, southeastern Mauritania<br><br>Transhumant pastoralists	n= 250	2·0% (0·7-4·6)	9.9	NR	NR	- Minimal distances between key infrastructure<br>- *Age<br>- Sex<br>- Marital status
Female sex workers (FSWs) / Men who have sex with men (MSM)/ Transgender individuals							

Willie (2021)<br><br>2016-2017	Cross-sectional, Papua New Guinea<br><br>FSWs, MSM, Transgender women (TGW)	n= 2,954<br><br>FSWs<br>n= 2,091<br><br>MSM and TGW<br>n= 863	FSWs:<br>- Port Moresby:<br>1,200 (100-2,400)<br>- Lae: 700 (100-1,200)<br>- Mt. Hagen: 200 (0-500)<br><br>MSM and TGW:<br>- Port Moresby:<br>1,000 (0-2,200)<br>- Lae: 1,200 (0-2,400)	FSWs:<br>0·6-3·6<br><br>MSM and TGW:<br>3-3·6	NR	NR	- TB-HIV co-infection (FSWs only)
PWUD							
Bernier (2020)<br><br>2016-2017	Cross-sectional, Abidjan, Ivory Coast<br><br>'Smoking spots'	n= 532	9·8% (7·5-12·7)	48·9	NR	NR	- *District<br>- Sex<br>- Age<br>- Living arrangement<br>- *Employment<br>- Family situation<br>- Educational level<br>- *History of TB<br>- Drug use practices<br>- History of imprisonment<br>- *TB-HIV co-infection
Minja (2021)<br><br>2016-2019	Cohort, Tanzania<br><br>Medication assisted treatment clinics	n= 901	Prevalence at enrollment:<br>2,553	NR	Incidence after 22 months:<br>2,925 (2,195-3,958)	12·3	- *Sex<br>- *Age<br>- *TB-HIV co-infection<br>- History of TB<br>- BMI<br>- Education<br>- Occupation<br>- Injection drug use<br>- Alcohol use<br>- Smoking status<br>- History of imprisonment
Nagot (2021)<br><br>2018	Cross-sectional, Hai Phong, Vietnam<br><br>People who inject drugs (PWID)	n= 885	HIV+ PWID<br>2·3% (1·0-4·5)<br><br>HIV- PWID<br>2·1% (0·8-4·2)	NR	NR	NR	- Age<br>- Gender<br>- *Marital status<br>- Employment<br>- *Homeless<br>- *Arrested over past 6 months

							- HIV care<br>- *Drug use behaviors<br>- Symptom screening
PLHIV							
Jarde (2022)	Meta-review,<br>Low and middle-income countries						
Gao (2013)<br><br>Up to 2011	Review & MA,<br>Global<br><br>(n= 21 countries)<br><br>TB/HIV co-infection<br><br>(HIV in TB+,<br>TB in HIV+)	47, n= 272,466<br><br>HIV from TB<br>n= 22,884<br><br>TB from HIV<br>n= 12,627<br><br>Unknown screening method<br>n= 236,955	Pooled estimate:<br>23·5% (20·9–26·1)<br><br>Range of prevalence:<br>2·9% (Iran) to 72% (Zambia)<br><br>Pooled Estimates:<br>- Africa:<br>31·3% (19·3–43·2); 17<br>- Asia:<br>17·2% (10–24·5); 9<br>- Latin America:<br>25·1% (19·3–30·8); 7<br><br>[additional data]<br>- Europe:<br>20·1% (13·8–26·4); 8<br>- USA:<br>14·8% (10·4–19·2); 6	NR	NR	NR	meta-regression;<br>- Year of publication<br>- Sex<br>- *Diagnosis method<br>- Screening type<br>- Setting<br>- Study type<br>- Sample size<br>- *Population prevalence (TB)<br>- *Population prevalence (HIV)
Gelaw (2019)<br><br>1990-2017	Review & MA,<br>sub-Saharan Africa<br><br>(n= 17 countries)<br><br>TB/HIV co-infection<br><br>(HIV in TB+)	68, n= 62,969<br>TB patients	Pooled estimate:<br>31·8% (27·8–36·1)<br><br>Range of prevalence:<br>6% (Ethiopia)<br>to 72% (Nigeria)<br><br>Pooled Estimates:<br>- Eastern SSA:<br>31·1% (25·4-37·5); 32<br>- Western SSA:<br>25·5% (19·7-32·3); 21<br>- Southern SSA:<br>43·7% (35·1-52·7); 12<br>- Central SSA:<br>41·3% (30·4-51·2); 3	NR	NR	NR	meta-regression;<br>- Form of TB<br>- Age<br>- Pop. Prevalence (TB)<br>- Research period<br>- Sample size<br>- *Geographical region<br>- *Pop. Prevalence (HIV)
	Review & MA,<br>China	15, n=12,127	Pooled estimate:<br>7·2% (4·2–12·3)	NR	NR	NR	Study base (population based or hospital based)

Gao (2010)	TB/HIV co-infection<br>(TB in HIV+,<br>HIV in TB+)		Range of prevalence:<br>0·5% to 35%				Gender<br>Target population (i.e. AIDS or HIV/AIDS)
Endalamaw (2019)	Review & MA,<br>Ethiopia<br><br>TB/HIV co-infection	13, n= 19,212	Pooled estimate:<br>23·4% (19·6–27·2)<br><br>Range of prevalence:<br>9·5% (Oromia) to 52·1% (Amhara)	NR	NR	NR	Geographic region<br>Known HIV status<br>Form of TB
Pourakbari (2019)	Review,<br>Iran<br><br>TB/HIV co-infection<br>(TB in HIV+,<br>HIV in TB+)	48, n= 21,388	Pooled estimate:<br>14·0% (12·0–15·0)<br><br>Range of prevalence:<br>0%)(Markazi) to 85% (Tehran)	NR	NR	NR	Injection drug use<br>Age < 40<br>Form of TB
Mixed 'at-risk' groups							
Bohlbro (2021)<br><br>2000-2020	Review & MA,<br>Global<br><br>Unspecified # of countries<br><br>'At-risk' groups: Refugees and immigrants, poor and marginalized, household contacts, drug users, health workers, PLHIV, prison inmates	144, n= 4,778,961 [at-risk groups]<br><br>53, n= 7,593,569 [general populations]	Overall at-risk groups:<br>1,401 (1,328-1,475)<br><br>Pooled estimates (selected):<br>- Refugees and immigrants: 257 (186–328); 14<br>- Poor and marginalized: 1,330 (1,165–1,495); 27<br>- Drug users:<br>1,157 (809–1,505); 2<br>- PLHIV:<br>9,092 (7,229–10,955); 22<br>- Prison inmates:<br>2,371 (1,983–2,759); 22	Overall at-risk groups:<br>12·5 (9·1-15·8)<br><br>- Refugees and immigrants:<br>1·1 (0·9–1·3)<br>- Poor and marginalized:<br>3·0 (2·0–3·9)<br>- Drug users:<br>11·9 (11·1–12·7)<br>- PLHIV:<br>19·7 (13·7–25·7)<br>- Prison inmates:<br>25·2 (11·6–38·8)	NR	NR	Rates and PRs available for ACF yield in at-risk groups and general populations by:<br>- World region<br>- High burden country classification (HBCs), and TB/HIV HBCs<br>- National incidence<br>- Study risk of bias<br>- Diagnostic/ screening method
Kranzer (2010)<br><br>1993-2009	Review & MA,<br>Global<br><br>Unspecified # of countries<br><br>Groups at high risk of HIV-associated TB in resource- limited settings	78, NR	Median values, ranges (selected groups):<br>- Congregate settings:<br>2·5%, 0·1-7·2; 21 (prisons in Africa, Asia, the Americas)<br>3·6%, 1·8–7·2; 7 (prisons in Sub-Saharan Africa)<br>2·3%, 1·2–5·0; 6 (mines)	NR	NR	NR	Analyses stratified by HIV status of the study population:<br>- Country prevalence (HIV)<br>- Availability of culture meta-regression;<br>- *Country prevalence (TB)<br>- *Symptom screening

			- Antiretroviral therapy and medical clinics:<br>8·2%, 1·4–24·7; 16<br>(Africa, Asia, the Americas)<br>8·6%, 3·6–24·7; 8<br>(sub-Saharan Africa)<br><br>- Voluntary counselling and testing services:<br>8·5%, 0·8–23·6; 10<br>- Prevention of mother-to-child transmission services:<br>2·3%, 2·1–3·5; 3				
Chimoyi (2020)<br>2000-2016	Review & MA, South Africa<br><br>National Strategic Plan for HIV, TB, STIs: key TB populations	49, n= 1,929	<u>Estimates (selected groups):</u><br>- Mineworkers: 1,300<br>- Informal settlements: 3,150<br>- Migrants and refugees: 36,000<br>- PLHIV: 3,000<br>- Inmates: 3,900	NR	- Mineworkers: 3,000<br>- Informal settlements: 4,500<br>- Migrants and refugees: 3,300<br>- PLHIV: 4,200	Relative risks:<br>- Mineworkers: 3·8<br>- Informal settlements: 5·8<br>- Migrants and refugees: 4·2<br>- PLHIV: 5·4	- Population attributable fractions
Abeid (2022)<br><br>2017-2020	Cross-sectional, Northwest Tanzania<br><br>Rural and mining communities	n= 144,707<br><br>Artisanal scale miners<br>n= 21,136<br><br>Female sex workers<br>n= 7,447	- Miners: 1·8% (1·6-2·0)<br>- FSWs: 1·7% (1·4-2·0)	Comparator general population *incidence*:<br>- Miners: 8.1<br>- FSWs: 7.7	NR	NR	- HIV referral care cascade by population groups
Dememew (2020)<br><br>2017-2018	Cross-sectional, Ethiopia<br><br>Nationally defined key and vulnerable populations for TB	n= 1,878<br><br>FSWs<br>n= 221<br><br>Miners or Internal Migratory Workers (IMWs)<br>n=237<br><br>Prison inmates<br>n= 1,112<br><br>IDPs<br>n= 102<br><br>Homeless	<u>Estimates (selected groups):</u><br>- FSWs:<br>2,817 (1,678-4,415)<br>- Miners or IMWs:<br>2,466 (1,468-3,869)<br>- Prison inmates:<br>794 (524-1,153)<br>- IDPs: 317 (80-1,756)<br>- Homeless: no cases detected	- FSWs: 17·2<br>- Miners or IMWs: 15<br>- Prison inmates: 4·8<br>- IDPs: 1·9<br>- Homeless: N/A	NR	NR	- Sex<br>- Age<br>- *Marital status<br>- *Educational status<br>- *History of TB<br>- *HIV status<br>- *Type of vulnerable population<br>- Diagnostic method

		n= 14					
Munteanu (2022)<br><br>2015-2017	Secondary data analysis (national surveillance), Romania<br><br>Vulnerable risk groups	n= 1,035<br><br>Inmates<br>n= 441<br><br>Homeless<br>n= 405<br><br>PWUD<br>n= 189	NR	NR	Average annual notification rate:<br><br>- Inmates: 542<br>- Homeless: 900<br>- PWUD: 630	Average (annualized)<br><br>- Inmates: 7·7<br>- Homeless: 12·8<br>- PWUD: 8·9	- *Sex<br>- *Age<br>- *History of TB<br>- *Pulmonary TB<br>- *Urban residence<br>- *TB-HIV co-infection<br>- *Diagnostic method
Kamenska (2019)<br><br>2014-2018	Cross-sectional, Ukraine<br><br>Key populations: Groups at risk of TB, and clients of harm reduction programs	n= 680,760	Overall amongst key populations screened: 343<br><br>- Homeless: 1,839-2,297<br>- MSM: 0-34<br>- PWID, Roma, ex-prisoner, sex workers<br>(data visually plotted)	3-13x increase in notification rates, when using active case finding strategies	NR	NR	- Yield by case finding strategy

PR= Prevalence ratio, IRR= Incidence rate ratio
MA= Meta-analysis
* = statistically significant (p<0·05)
† Unless otherwise stated, comparators (PR/IRR) refer to corresponding general population prevalence or incidence rates (typically at the national level, per 100,000 persons). This information was not always specified in source material. Reference values did not always correspond to the year in which studies were conducted. Comparators were used as presented by source authors, or where absent were calculated manually as permitted.
‡ The comparison groups used in studies differed widely between studies. Numbers provided by authors for regional estimates were sometimes regional averages, while other times they were non-indigenous TB rates within those regions.

5. Supplementary Figure 1: Forest plot of TB prevalence estimates among vulnerable populations, per 100,000

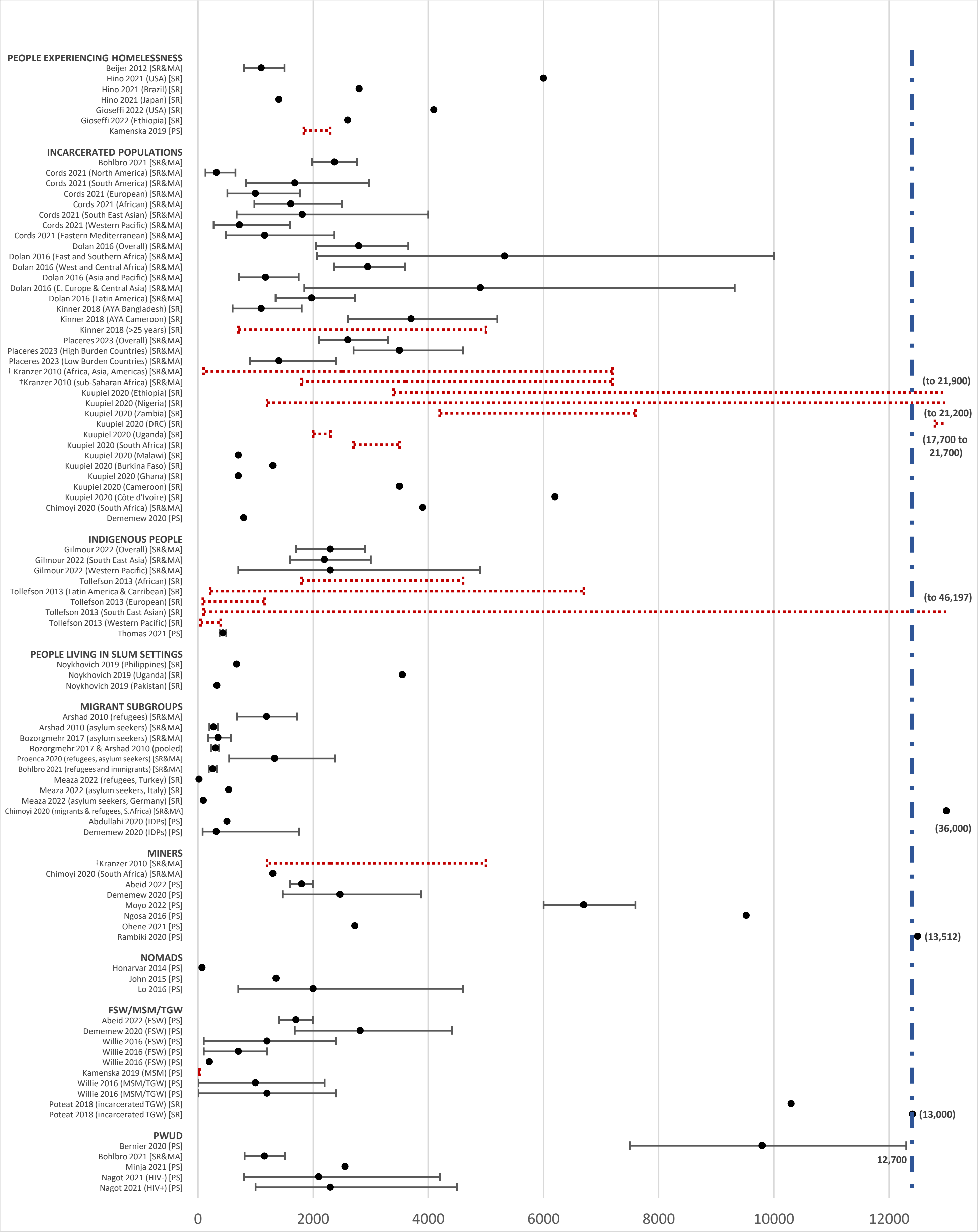

[Supplementary Figure 1]
Point estimates (in black) are reported with 95% confidence intervals, as available.
Red dashed lines represent absolute ranges of crude estimates. The vertical line signifies a break in the x-axis (outlier data points have been brought forward from outside the field of view).

[SR&MA]= systematic review and meta-analysis, [SR]= systematic review, [PS]= primary/database study.

† Estimates are median values (with ranges).

6. Supplementary Figure 2: Forest plot of TB prevalence estimates among PLHIV

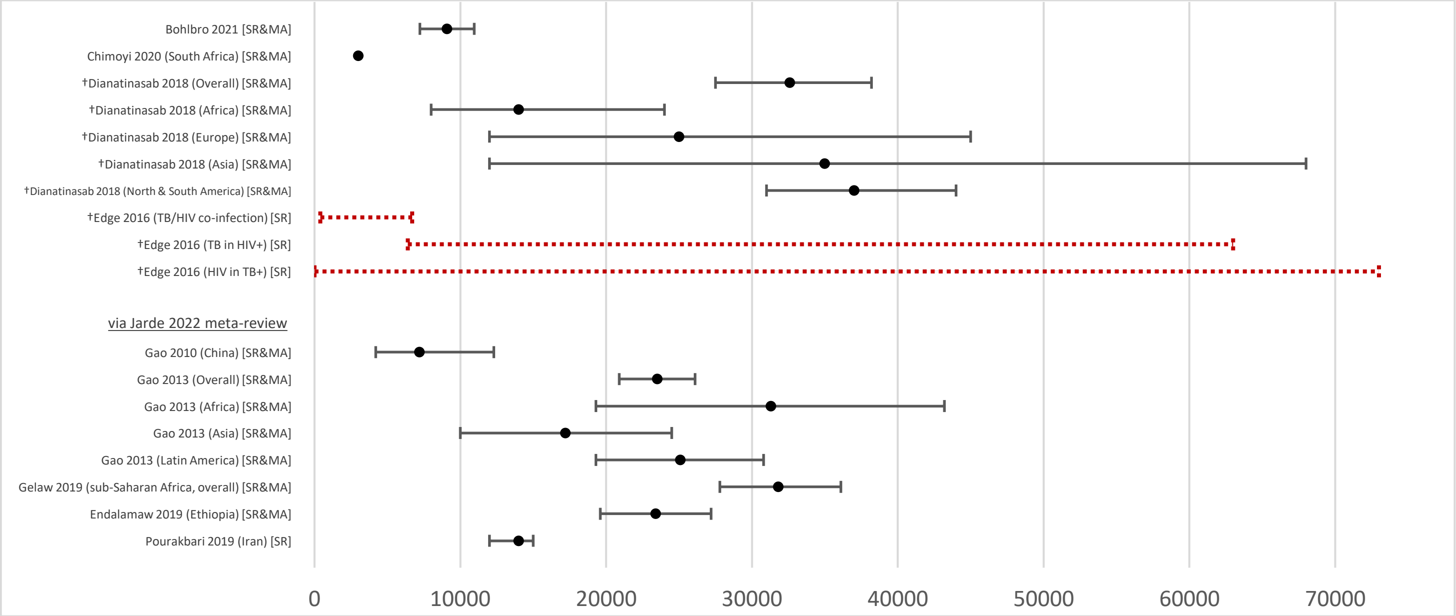

Data is per 100,000. Point estimates (in black) are reported with 95% confidence intervals, as available. Red dashed lines represent absolute ranges of crude estimates.

[SR&MA]= systematic review and meta-analysis, [SR]= systematic review.

† Review on incarcerated populations.

Note: Figure is representative of studies where PLHIV or TB/HIV co-infection was considered a main group of interest (does not include studies documenting HIV status as clinical subdata).

7. Supplementary Figure 3: Forest plot of TB incidence estimates among vulnerable populations

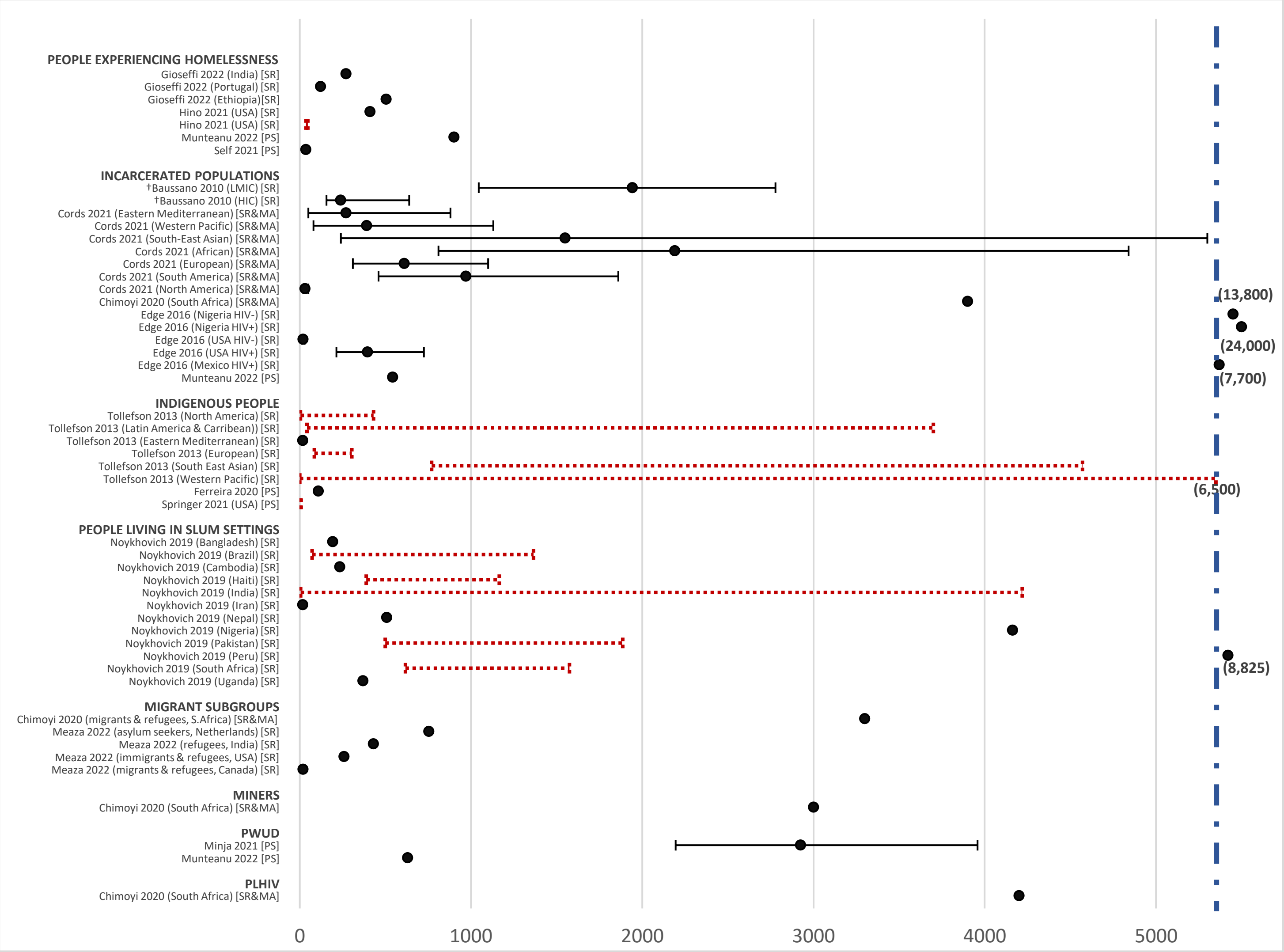

Data is per 100,000 person-years. Point estimates (in black) are reported with 95% confidence intervals, as available. Red dashed lines represent absolute ranges of crude estimates. The vertical line signifies a break in the x-axis (outlier data points have been brought forward from outside the field of view).

[SR&MA]= systematic review and meta-analysis, [SR]= systematic review, [PS]= primary/database study.

† Estimates are median values (with interquartile range).

8. Supplementary Maps: Availability of Studies Reporting Data on the Prevalence and/or Incidence of TB Disease in Vulnerable Population Groups between 2010 and 2023, by country and background TB burden.

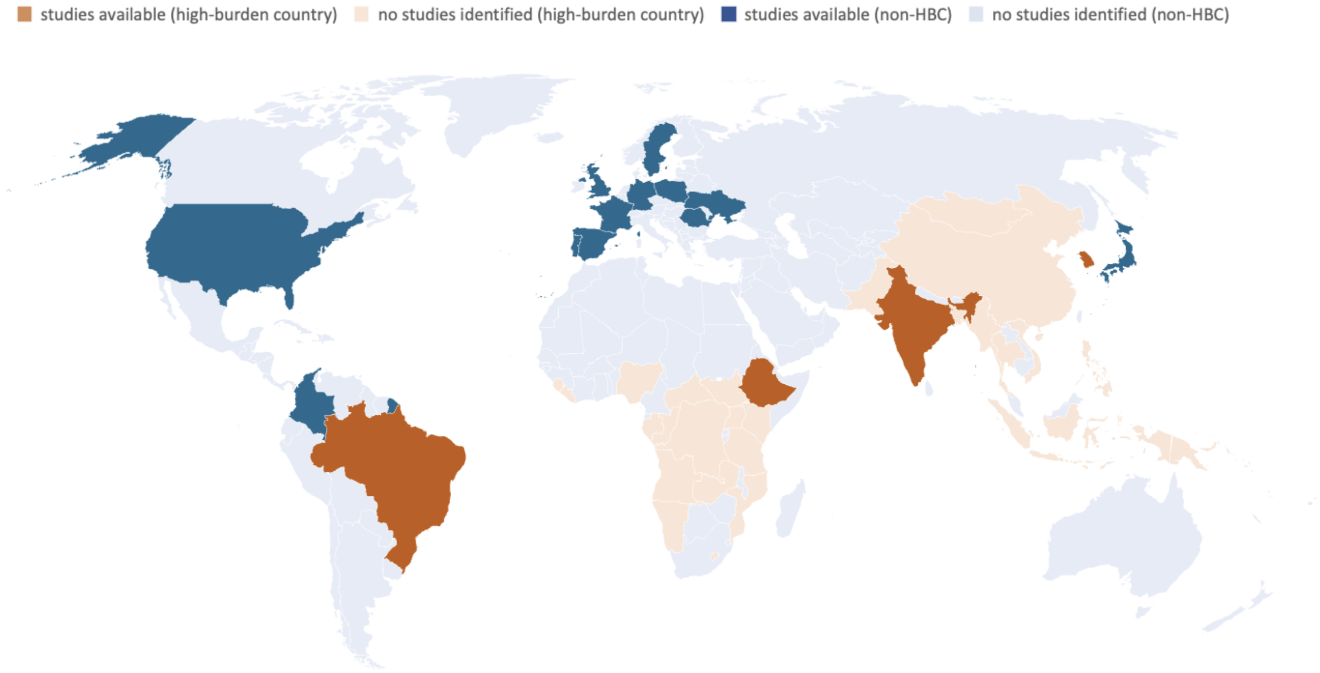

I. People experiencing homelessness

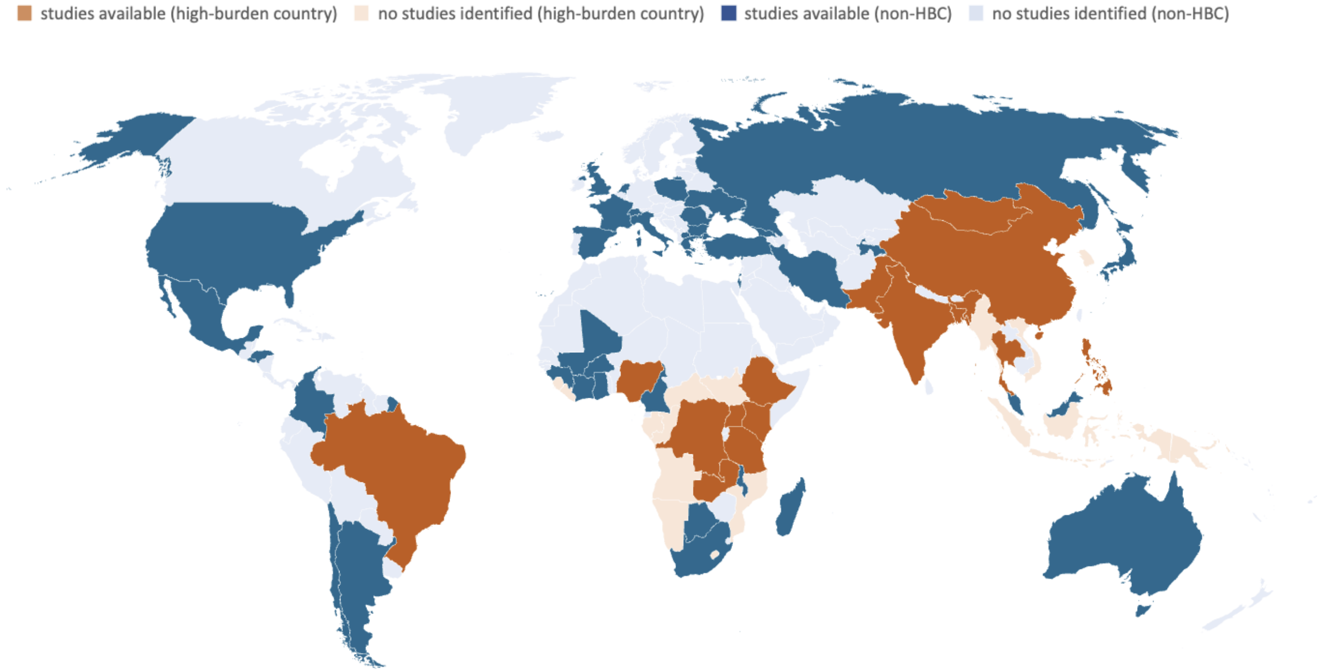

II. Incarcerated populations*
*Map not inclusive of countries listed in Dadu et al. (2020); i.e., 42/53 (79%) of WHO European Region countries reporting new and relapse TB cases in the civilian or penitentiary sectors at least once between 2014-2018.

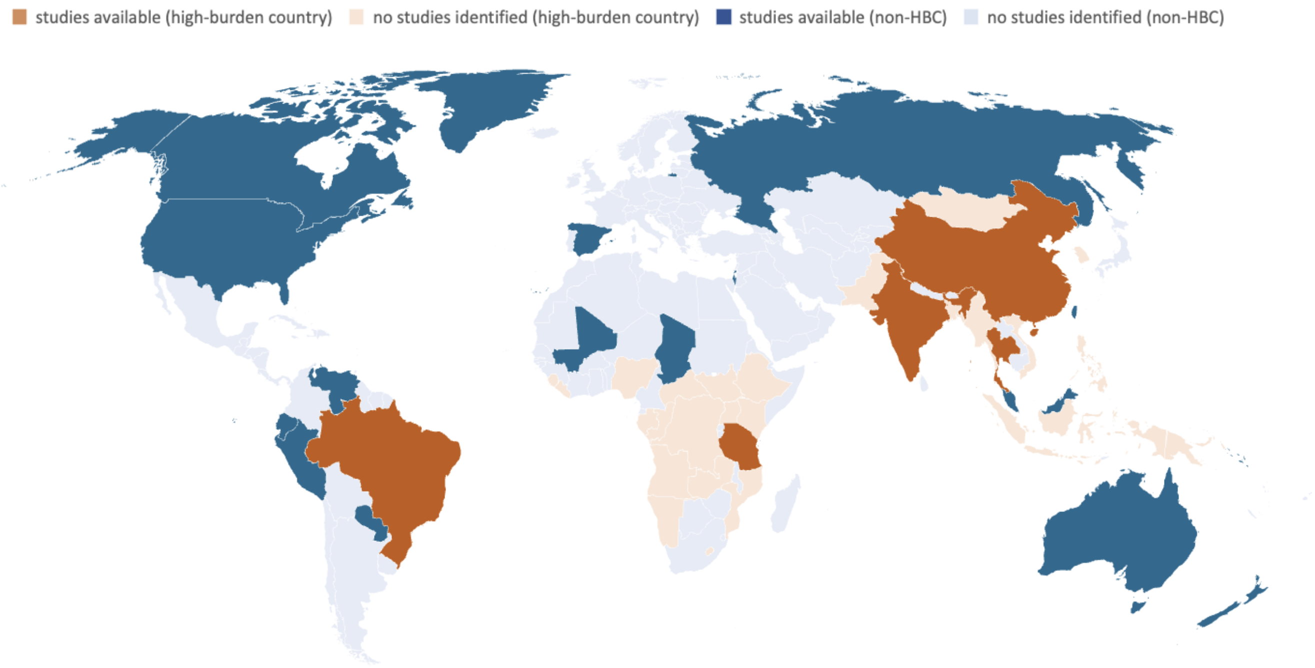

III. Indigenous populations

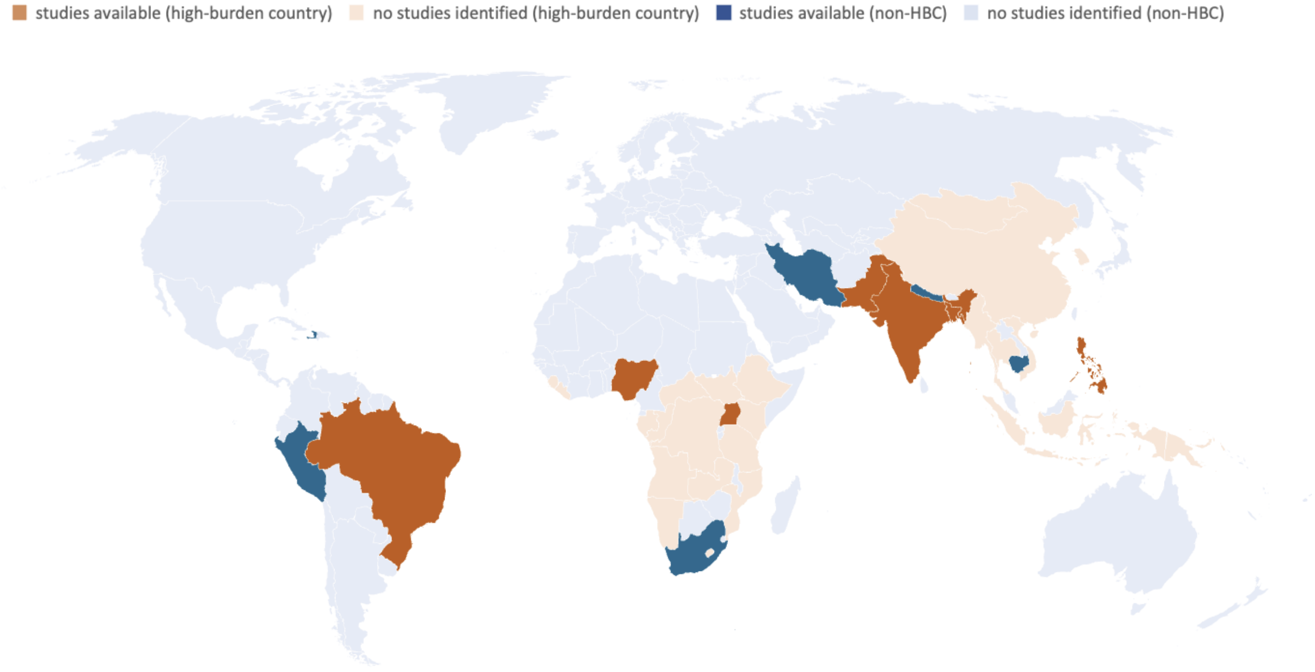

IV. People living in slum settings

studies available (high-burden country) no studies identified (high-burden country) studies available (non-HBC) no studies identified (non-HBC)

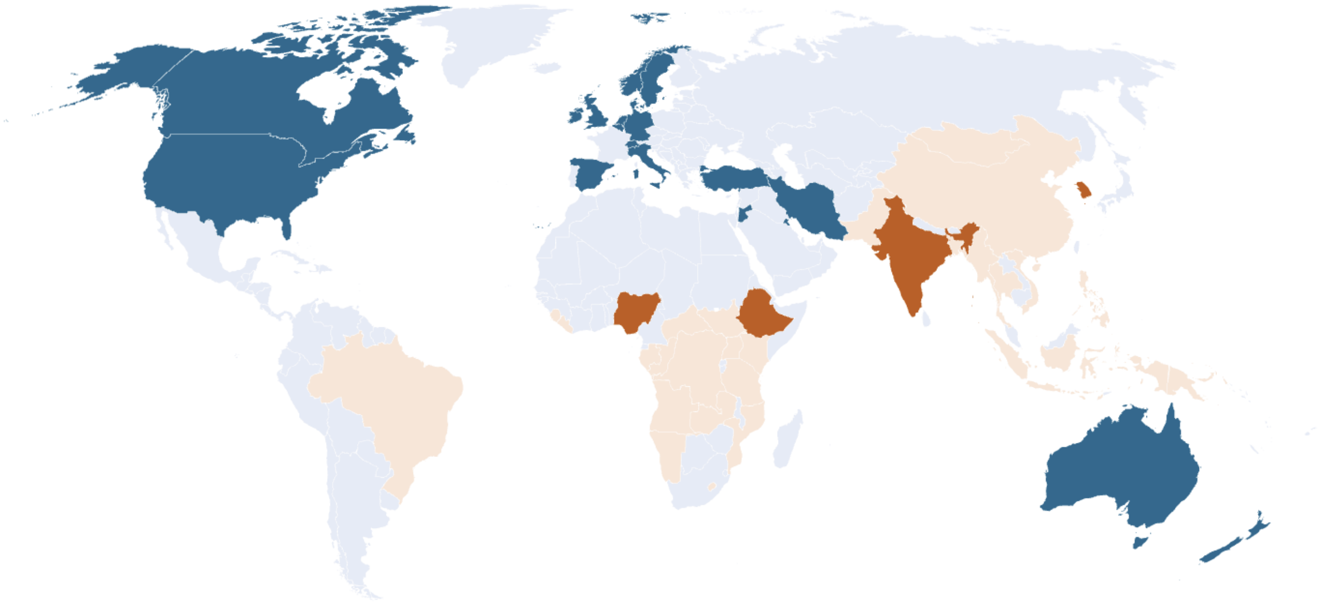

V. Refugees, asylum seekers, internally displaced persons
(by host country / location of studies)

studies available (high-burden country) | no studies identified (high-burden country) | studies available (non-HBC) | no studies identified (non-HBC)

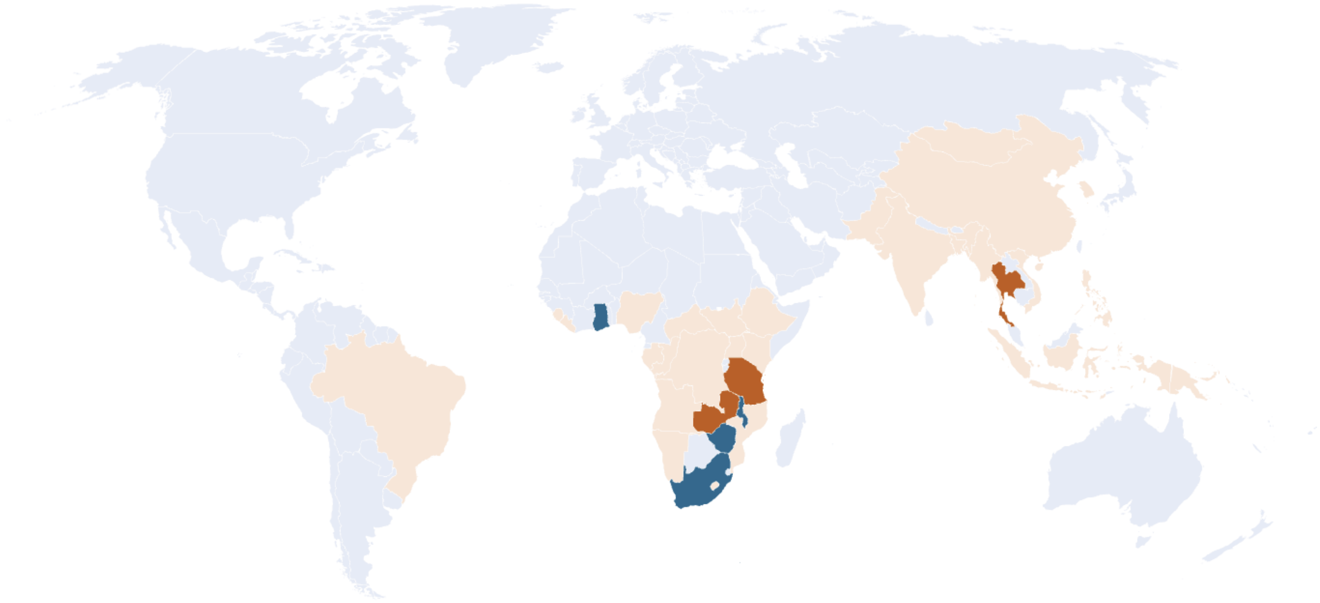

VI. Miners

studies available (high-burden country) | no studies identified (high-burden country) | studies available (non-HBC) | no studies identified (non-HBC)

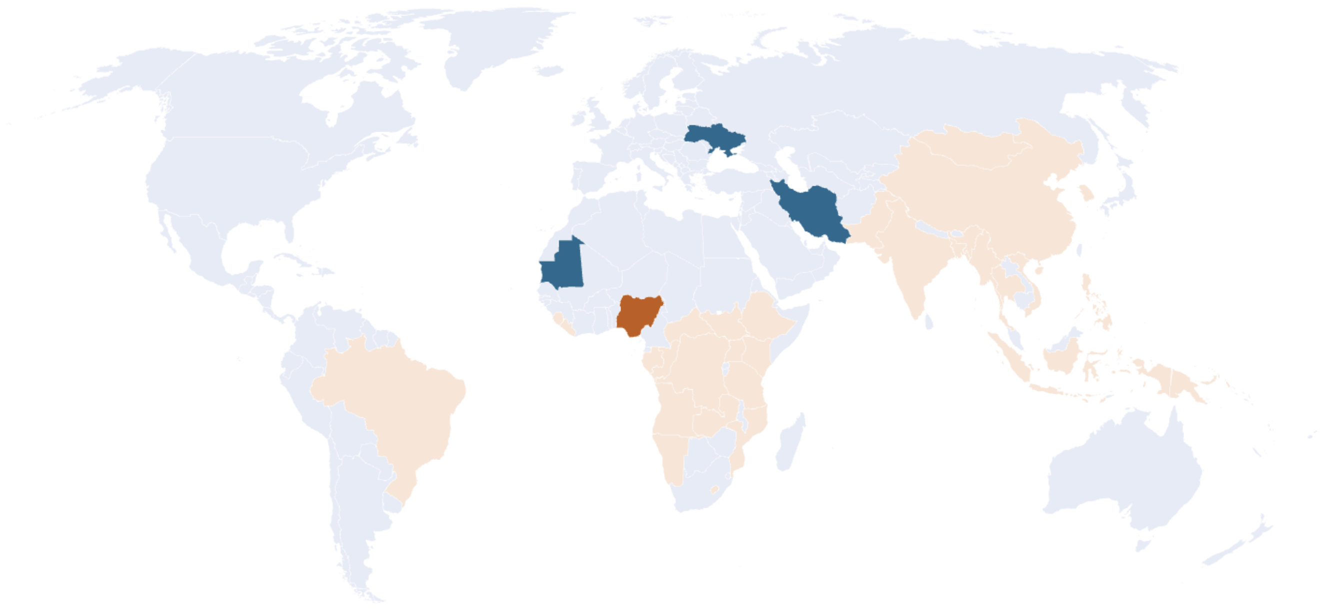

VII. Nomadic populations

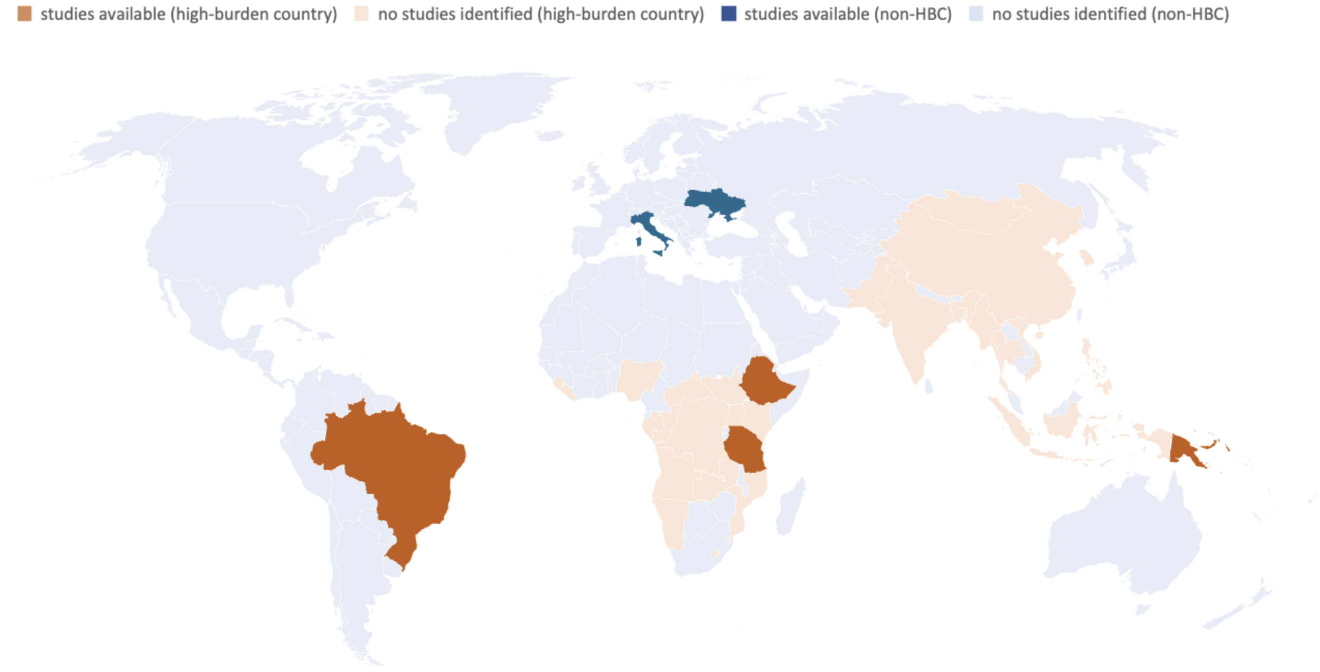

VIII. Female sex workers, IX. Men who have sex with men, and X. Transgender individuals

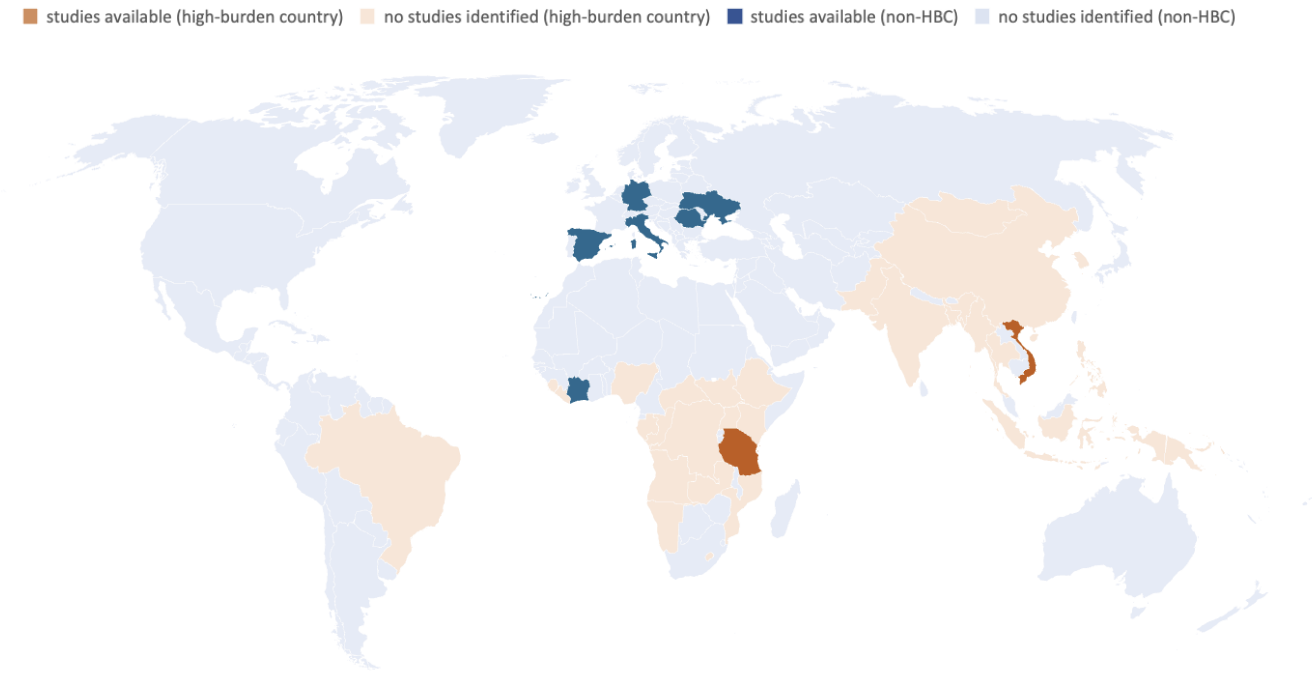

XI. People who use drugs

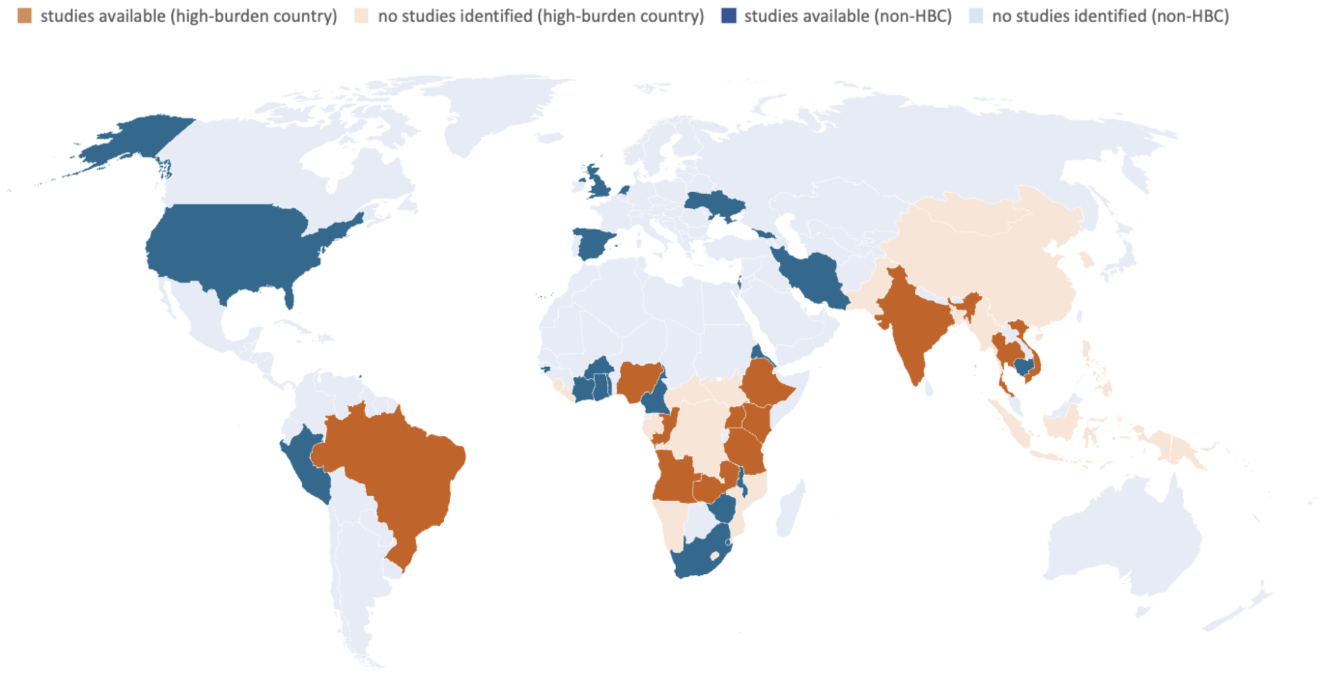

XII. People living with HIV*
*Map represents only studies where PLHIV or TB/HIV co-infection was considered a main group of interest (does not include studies documenting HIV status as clinical subdata).

9A. AMSTAR 2 Assessments of Included Reviews

Study ID	Q1	Q2 * †	Q3	Q4 *	Q5	Q6	Q7 ‡	Q8	Q9 *	Q10	Q11*	Q12	Q13 *	Q14	Q15*	Q16	Overall
**1. Arshad 2010**	Y	N/A	Y	PY	Y	Y	N	Y	Y	N	Y	N	N	Y	Y	Y	**Low**
**2. Baussano 2010**	Y	N/A	N	PY	Y	Y	N	PY	Y	N	N/A	N/A	Y	Y	N/A	Y	**Moderate**
**3.Beijer 2012**	Y	N	N	PY	N	Y	N	Y	N	N	Y	N	N	Y	N	Y	**Low**
**4.Bohlbro 2021**	Y	Y	Y	PY	Y	Y	N	Y	Y	N	Y	Y	Y	Y	Y	Y	**High**
**5. Bozorgmehr 2017**	Y	Y	Y	PY	Y	N	N	Y	N	Y	Y	Y	N	Y	N	Y	**Critically low**
**6. Chimoyi 2020**	N	N	Y	N	Y	N	N	Y	N	N	Y	N	Y	Y	N	Y	**Critically low**
**7. Cords 2021**	Y	Y	Y	PY	Y	Y	N	PY	Y	N	Y	N	Y	Y	N	Y	**Low**
**8. Dianatinasab 2018**	Y	N	N	PY	Y	Y	N	Y	PY	N	N	Y	N	Y	Y	Y	**Critically low**
**9. Dolan 2016**	Y	N	N	PY	Y	Y	N	N	N	N	N	N	N	N	N	Y	**Critically low**
**10. Edge 2016**	Y	Y	N	PY	Y	N	N	Y	Y	N	NA	N/A	N	Y	N/A	Y	**Low**
**11. Gilmour 2022**	Y	Y	Y	PY	Y	Y	N	N	Y	N	Y	N	N	Y	Y	Y	**Low**
**12. Gioseffi 2022**	N	N	N	PY	Y	N	N	PY	Y	N	N/A	N/A	N	N	N/A	Y	**Critically low**
**13. Hino 2021**	Y	N	N	N	Y	N	N	Y	N	N	N/A	N/A	N	Y	N/A	N	**Critically low**
**14. Jarde 2022**	Y	Y	Y	Y	Y	Y	Y	Y	Y	N	N/A	N/A	Y	Y	N/A	Y	**High**
**15. Kinner 2018<br>Re-analysis of Dolan 2016**	N/A	N/A	N/A	N/A	N/A	N/A	N/A	N	N	N	Y	N	N	Y	N	Y	**Critically low**
**16. Kuupiel 2020**	Y	PR	Y	PY	Y	Y	N	Y	PY	N	N/A	N/A	N	N	N/A	Y	**Low**
**17. Kranzer 2010**	Y	N/A	N	N	Y	Y	N	Y	N	N	N/A	N/A	N	Y	N/A	Y	**Low**
**18. Meaza 2022**	N	Y	Y	PY	Y	N	N	Y	Y	N	N/A	N/A	N	Y	N/A	Y	**Low**
**19. Noykhovich 2019**	Y	N	N	PY	N	N	N	PY	PY	N	Y	N	Y	N	Y	Y	**Low**

**20. Placeres 2023**	Y	PY	Y	PY	Y	Y	N	N	Y	N	Y	Y	Y	Y	N	Y	**Low**
**21. Poteat 2018**	Y	N	N	PY	N	N	N	Y	N	N	N/A	N/A	N	N	N/A	Y	**Critically low**
**22. Proenca 2020**	Y	Y	N	PY	Y	Y	N	Y	N	N	N	N	N	N	N	Y	**Critically low**
**23. Tollefson 2013**	Y	N	Y	PY	Y	Y	N	Y	N	N	N/A	N/A	N	Y	N/A	Y	**Critically low**

Y: Yes
PY: Partial Yes
N: No
N/A: Not applicable
* Critical Domain
† Reporting standards for systematic reviews have developed rapidly over the past decade. The expectation of a protocol was not universally recognized until the publication of the PRISMA reporting guidelines in 2009. Allowing for 2 years of uptake, we assigned 'N/A' to Q2 (explicit statement of protocol/registration) to any systematic review published prior to 2012.
‡ We did not consider Q7 (Did the review authors provide a list of excluded studies and justify the exclusions?) as a critical domain for assessment.

AMSTAR 2: a critical appraisal tool for systematic reviews that include randomised or nonrandomised studies of healthcare interventions, or both

Questions (listed short-form, without underlying criteria):
1. Did the research questions and inclusion criteria for the review include the components of PICO?
2. Did the report of the review contain an explicit statement that the review methods were established prior to the conduct of the review and did the report justify any significant deviations from the protocol?
3. Did the review authors explain their selection of the study designs for inclusion in the review?
4. Did the review authors use a comprehensive literature search strategy?
5. Did the review authors perform study selection in duplicate?
6. Did the review authors perform data extraction in duplicate?
7. Did the review authors provide a list of excluded studies and justify the exclusions?
8. Did the review authors describe the included studies in adequate detail?
9. Did the review authors use a satisfactory technique for assessing the risk of bias (RoB) in individual studies that were included in the review?
10. Did the review authors report on the sources of funding for the studies included in the review?
11. If meta-analysis was performed did the review authors use appropriate methods for statistical combination of results?
12. If meta-analysis was performed, did the review authors assess the potential impact of RoB in individual studies on the results of the meta-analysis or other evidence synthesis?
13. Did the review authors account for RoB in individual studies when interpreting/ discussing the results of the review?
14. Did the review authors provide a satisfactory explanation for, and discussion of, any heterogeneity observed in the results of the review?
15. If they performed quantitative synthesis did the review authors carry out an adequate investigation of publication bias (small study bias) and discuss its likely impact on the results of the review?
16. Did the review authors report any potential sources of conflict of interest, including any funding they received for conducting the review?

The studies were judged to be of high quality if the included reviews did not contain any major flaws in critical domains (zero or one flaw allowed in a non-critical domain); of moderate quality if there were no flaws in the critical domains, but more than one flaw was identified in the non-critical domains; low quality if it had one major flaw in a critical domain; and critically low quality if it had more than one major flaw in a critical domain.

Shea BJ, Reeves BC, Wells G, Thuku M, Hamel C, Moran J, Moher D, Tugwell P, Welch V, Kristjansson E, Henry DA. AMSTAR 2: a critical appraisal tool for systematic reviews that include randomised or non-randomised studies of healthcare interventions, or both. BMJ. 2017 Sep 21;358:j4008.

9B. Modified JBI Critical Appraisal Checklist for Primary/Database Studies

Study ID	Was the sample frame appropriate to address the target population?	Were study participants recruited in an appropriate way?	Was the sample size adequate?	Were the study subjects and the setting described in detail?	Was the condition measured in a standard, reliable way for all participants?	Was the response rate adequate, and if not, was the low response rate managed appropriately?	Was there appropriate statistical analysis?	Were confounding factors identified?	Were strategies to deal with confounding factors stated?
**1. Abdullahi 2020**	N	Y	UC	Y	Y	UC	Y	N	N
**2. Abeid 2022**	Y	Y	UC	Y	Y	UC	Y	N	N
**3. Bernier 2020**	Y	Y	N	Y	Y	Y	Y	Y	Y
**4. *Dadu 2021**	Y	N/A	N/A	Y	Y†	Y	Y	N	N
**5. Dememew 2020**	Y	Y	UC	Y	Y	Y	Y	Y	Y
**6. *Ferreira 2020**	Y	N/A	N/A	Y	Y†	N/A	Y	N	N
**7. Honarvar 2014**	UC	UC	UC	Y	Y	Y	N	N	N
**8. John 2015**	UC	UC	UC	Y	Y	UC	Y	N	N
**9. Kamenska 2019**	UC	UC	UC	Y	Y	UC	N	N	N
**10. Lo 2016**	UC	Y	Y	Y	N	Y	Y	Y	Y
**11. Minja 2021**	UC	Y	Y	Y	Y	Y	Y	Y	Y
**12. Moyo 2022**	Y	Y	Y	Y	Y	Y	Y	Y	N
**13. *Munteanu 2022**	Y	N/A	N/A	Y	Y†	N/A	Y	Y	Y
**14. Nagot 2021**	UC	N	N	Y	Y	Y	Y	Y	Y
**15. Ngosa 2016**	Y	Y	Y	Y	Y	Y	Y	Y	Y
**16. Ohene 2021**	N	N	UC	Y	Y	UC	Y	Y	Y
**17. Rambiki 2020**	UC	N	Y	Y	Y	Y	Y	Y	Y

**18. *Self 2021**	Y	N/A	N/A	Y	Y†	N/A	N	N	N
**19. *Springer 2021**	Y	N/A	N/A	Y	Y†	N/A	Y	Y	Y
**20. Thomas 2021**	Y	Y	N	Y	Y	Y	Y	Y	Y
**21. Willie 2021**	UC	N	N	Y	Y	UC	Y	N	N

Y: Yes
N: No
UC: Unclear
N/A: Not applicable
* Studies based on nationally notified cases, † Case information was extracted from national registries, therefore it is expected that a standardized case definition/criteria has been applied – although further details have not been provided.
